# Understanding Degradation in Single-Crystalline Ni-Rich Li-Ion Battery Cathodes

**DOI:** 10.1021/acs.chemrev.5c00330

**Published:** 2025-10-09

**Authors:** Matthew J. W. Ogley, Beth I. J. Johnston, David S. Hall, Louis F. J. Piper

**Affiliations:** † WMG, 2707 University of Warwick, Coventry CV4 7AL, U.K.; ‡ The Faraday Institution, Quad One, Harwell Campus, Didcot OX11 0RA, U.K.; § Department of Energy and Petroleum Engineering, 56627 University of Stavanger, 4021 Stavanger, Norway

## Abstract

The growing demand for ever-higher-energy-density Li-ion batteries has accelerated the development of Ni-rich transition metal (TM) oxide cathodes. Despite their potential, unsolved degradation mechanisms continue to limit their practical capacity and cycle life. Single-crystalline morphologies have emerged as a promising solution, offering superior mechanical and structural stability compared to polycrystalline cathodes. Nevertheless, degradation still occurs, driven by atomic-scale instabilities, interfacial side reactions, and particle-level mechanical strain. To address these challenges, this review systematically examines cathode development from the atomic to cell level and provides critical insight into how different material design strategies can enhance long-term performance.

## Graphical abstract

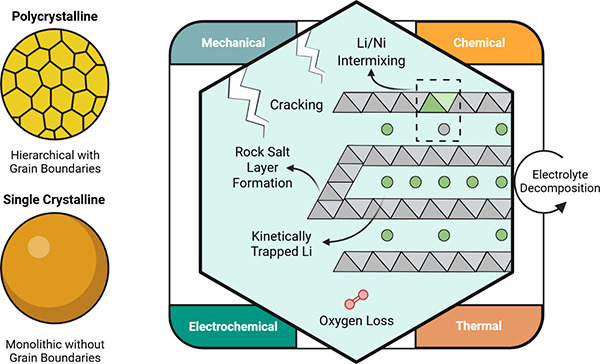

## Introduction

Both single-crystalline (SC) and Ni-rich layered transition metal (TM) oxide cathodes have emerged as critical areas of focus in the energy storage industry due to their high energy density and cycle life. This review examines the chemical and morphological properties of these materials and serves as a detailed resource that can be used by new and experienced researchers alike. We provide an in-depth analysis of synthesis-structure-performance relationships, cell fabrication, cycling protocols, degradation mechanisms, and advanced strategies for enhancing performance. By consolidating key advances, ongoing challenges, and future directions, this article aims to bridge fundamental insights with industrial and commercial applications in the hopes of accelerating innovation in high-energy-density SC Li-ion battery cathodes.

### A Brief History of Li-ion Battery Development

In the early 1970s, there was a growing interest in using Li metal as a lightweight, energy-dense, anode material. Thus, there was a need to develop a suitable cathode material that could accommodate Li-ions. A successful candidate would have to be stable of over a wide range of Li stoichiometries, have high electronic conductivity, possess high solid-state diffusivity of the Li-ions, and be insoluble in nonaqueous electrolyte solutions. By the mid-1970s, Li_*x*_TiS_2_ had been explored by chemical lithiation (through reaction with *n*-butyllithium in ammonia) and was a promising intercalation host for alkali metals.^1–3^ In 1974, M. Stanley Whittingham published the first demonstration of Li_*x*_TiS_2_ being a viable electrochemical intercalation electrode.^4^ While working at Exxon, Whittingham filed a patent for this invention in 1975,^5^ published a description of a rechargeable Li-based battery in 1976,^6^ and presented prototype 45 Wh Li/TiS_2_ cells at the Chicago electric vehicle (EV) show in 1977.^7,8^ Around this time, Rudi Haering, Klaus Brandt, and James Stiles began exploring MoS_2_ as an alternative chalcogenide that addressed the complex and costly synthesis of TiS_2_.^9–11^ AA-sized Li/MoS_2_ cells entered commercial production in 1988 and were the first rechargeable Li-based batteries. However, within months, safety incidents caused by the unstable Li metal anode prompted a widespread recall, thereby driving the industry to shift toward alternative electrode materials.^12^

In 1980, John Goodenough discovered that LiCoO_2_ and LiNiO_2_ could undergo electrochemical delithiation at twice the open-circuit voltage (OCV) of the TM chalcogenides.^13–15^ Though both materials were patented soon after in 1982,^16^ the commercialization of TM oxides was slowed by its initial combination with Li metal anodes and the thermodynamic instability of the then-available electrolytes. Addressing the former, in 1983, Akira Yoshino developed a cell with a LiCoO_2_ cathode and polyacetylene anode.^17^ Owing to its poor volumetric energy density, polyethylene was quickly replaced by carbonaceous materials in 1985.^17^ Thereafter, seminal work by Jeff Dahn outlined the positive impact of including ethylene carbonate (EC) in the electrolyte due to its ability to form a stable solid-electrolyte interphase (SEI).^18^ These advancements enabled Sony to commercialize the first TM-oxide Li-ion battery in 1991, marking the beginning of the modern Li-ion battery era.

As the demand for larger-format applications, such as EVs, grew, so did the focus on safety. This led researchers to incorporate redox-inactive elements such as Al and Mn into the TM oxide structure to create NCA (LiNi_*x*_Co_*y*_Al_1‑x‑y_O_2_) and NMC (LiNi_*x*_Co_*y*_Mn_1‑x‑y_O_2_). The latter, which will be focused on in this review, has seen many successive iterations, progressing from NMC111 (Ni:Mn:Co = 1:1:1) to NMC811(8:1:1), with NMC111 offering greater thermodynamic stability and NMC811 offering superior specific capacity.^19,20^

Outside academia, the commercial adoption of NMC was delayed by extensive legal disputes over the rights to intellectual property. The most notable conflict erupted when BASF, after acquiring Argonne National Laboratory’s NMC patents, sued Umicore for infringement (which had licensed similar technology from 3M). Ultimately, to avoid prolonged litigation risks and address both parties’ mutual need for market stability, the legal battle concluded with a cross-licensing agreement in 2021 strengthening the American and European position against a burgeoning Korean and Chinese EV battery market.

### State of the Art NMC811

Today, NMC development is motivated by the push toward increasingly Ni-rich compositions. This reflects the need to maximize energy density and minimize the use of Co-based precursors. As of early 2025, the current state-of-the-art Li-ion battery contains polycrystalline (PC) NMC811, a Ni-rich layered transition metal (TM) oxide of the form LiTMO_2_ (where TM = Ni, Mn, Co). It possesses a theoretical specific capacity of 275 mAh g^–1^ and an average discharge voltage of 3.8 V vs Li^+^/Li.^21^ NMC811 adopts the α-NaFeO_2_ structure, which belongs to the rhombohedral crystal system with space group R3̅m.^22^ It consists of a cubic close-packed (ccp) array of O anions, with octahedrally coordinated Li^+^ and TM cations occupying the *3a* and *3b* Wyckoff positions, respectively.^23^ Li-ion diffusion occurs through the *ab*-plane via adjacent face-sharing tetrahedral sites and is enabled by a charge compensation mechanism that prevents polarization of the electrodes.^24^ Recently, there has been success in utilizing condensed matter physics frameworks to describe the redox mechanism of Ni-rich TM oxide cathodes through the formation and elimination of ligand holes (L).^25–28^ Unfortunately, that same redox mechanism was identified as the origin of O loss,^29^ layered to rock-salt phase transformations,^30^ and an increased susceptibility to thermal runaway.^31,32^ To contextualise these degradation mechanisms and identify their origins, this review provides a comprehensive overview of the Li-ion battery development process, wherein a thorough understanding of the interplay between mechanical, chemical, and electrochemical degradation can inform the discovery of material and design solutions.

### Review Scope

Of particular focus in this article is the cathode particle morphology. Traditionally, LiTMO_2_ cathodes are polycrystalline and can be described as spherical secondary particles composed of smaller individual primary particles. While this hierarchical structure is cost-effective, scalable, and offers the lowest surface area-to-bulk ratio, it is mechanically fragile, especially during repeated charge–discharge cycles. Grain boundary misalignment in PC particles exacerbates this issue and leads to cracking which exposes new surfaces to the electrolyte, thereby promoting parasitic side reactions that reduce performance. In contrast, SC morphologies, characterized by larger more uniformly sized primary particles, exhibit minimal grain boundary stress.^33^ This improves mechanical integrity which translates to reduced particle cracking, better Li-ion diffusion, and improved cycling performance under ever-more demanding conditions. Academically, SC particles also serve as a clean model system that enables the precise investigation of fundamental mechanisms which are unobscured by grain boundary effects.

M. Stanley Whittingham’s TiS_2_ cathodes were originally large single crystals,^5,34^ and the recent revival of this morphology with the development of the ’million-mile’ NMC532 battery^35^ offers a promising opportunity to overcome the limitations of conventional PC cathodes. Provided that SC Ni-rich NMC exhibits a crystal size below 3.5 μm, bulk degradation (i.e., fragmentation) does not occur.^36^ However, well-controlled SC LiTMO_2_ particle synthesis is challenging, as is developing industrially scalable production methods. Additionally, electrode formulations, including slurry mixing, coating, and calendaring, must all be carefully tailored in order to maintain the inherent advantages of SC cathodes. As such, understanding each of the processing steps is crucial. Furthermore, the same electrochemical considerations such as voltage cutoffs, C-rates, and electrolyte formulations apply to both morphologies. This outlines the holistic approach needed to develop these materials,

This review integrates atomic- and particle-level insights with discussions on material synthesis, electrode formulation, electrochemical performance, and degradation mechanisms. Special attention is given to morphological control, and advanced characterization techniques. These include ex-situ, in situ and operando methods, together with computational modeling, to elucidate fundamental phenomena that govern cathode performance. These studies reveal that while SC Ni-rich NMC cathodes show improved resistance to mechanical cracking, high voltage and fast cycling still induce structural defects such as Li-ion heterogeneity and plane gliding. O_2_ loss driven by thermodynamic instability leads to surface reconstruction that hampers Li-ion diffusion, causing crystallographic fatigue and kinetic trapping of Li, which together contribute to capacity fade.

Due to its advanced commercial development, widespread use in current-generation Li-ion batteries, and the extensive availability of experimental data, this review focuses on SC-NMC811 as a representative Ni-rich layered oxide. Its stoichiometry (80% Ni) makes it an ideal benchmark for understanding degradation mechanisms that are broadly relevant across Ni-richer and Ni-poorer NMC chemistries. Specifically, the insights derived from SC-NMC811 are transferable to emerging compositions e.g. NMC9055 and LiNiO_2_, as many degradation phenomena such as O_2_ loss, surface reconstruction, and crystallographic fatigue share similar origins. Understanding and controlling these degradation pathways is key to improving energy density and cycle life. Therefore, this review concludes with an overview of current strategies used to mitigate performance fade, with the aim of guiding future cathode design.

## Atomic- and Particle-Level Properties

Understanding the atomic- and particle-level properties of Ni-rich LiTMO_2_ cathodes is crucial for optimizing their performance in high-energy-density Li-ion batteries. While Li content influences theoretical specific capacity and energy density, atomic and particle-level behavior determines how reversibly the available capacity can be utilized. It is therefore important to investigate these properties and explain how they relate to the overall electrochemical performance.

### Crystallographic Transformations in Intercalation Compounds

Ni-rich LiTMO_2_ cathodes possess an O3-type α-NaFeO_2_-type layered structure with R3̅m space group symmetry. The “O3” notation was introduced by Claude Delmas in 1980 to classify layered TM oxides containing an octahedrally coordinated charge carrier (Li), with an O sublattice that adopts an ABC stacking sequence.^23^ In this notation, the letter indicates the coordination environment of the alkali ion: O for octahedral and P for prismatic. In some cases, additional primes (′) are added to distinguish between structurally similar but symmetry-distinct phases. For example, the O′ phase retains the octahedral coordination of the alkali ion but crystallizes in a monoclinically distorted version of the original O3 phase. Then, the subsequent number refers to the number of repeating TM-O_2_ oxide layers in the unit cell (i.e., how many TM–O_2_ slabs are stacked along the *c*-axis). in an O3-NMC, (e.g., the leftmost structure in Figure 1) the Li^+^ cations occupy octahedral sites between the O anions, and there are three TM-O_2_ layers in each unit cell.^23^ Furthermore, many other Delmas notations exist that can describe the various structures that exist throughout the layered oxides. For example, the O2-type structure features octahedrally coordinated Li ions but with an AB O stacking sequence, whereas the P2-type structure exhibits the same AB stacking but with prismatically coordinated Li ions. Under extreme delithiation, O1-type structures possessing a vertically aligned TMO_2_ stacking pattern can emerge. Changes in the stacking sequence is detrimental for Li-ion battery performance and in the case of the O3 → O1 transition, the O1 phase is more vulnerable to O_2_ loss and rock salt formation.^37,38^ Fortunately, in SC-NMC811, the deleterious O3 → O1 phase transition is not observed under conventional cycling conditions.^21^ It can only be detected by techniques sensitive to long-range order after a prolonged high-voltage high-temperature hold.^22^

**Figure 1:**
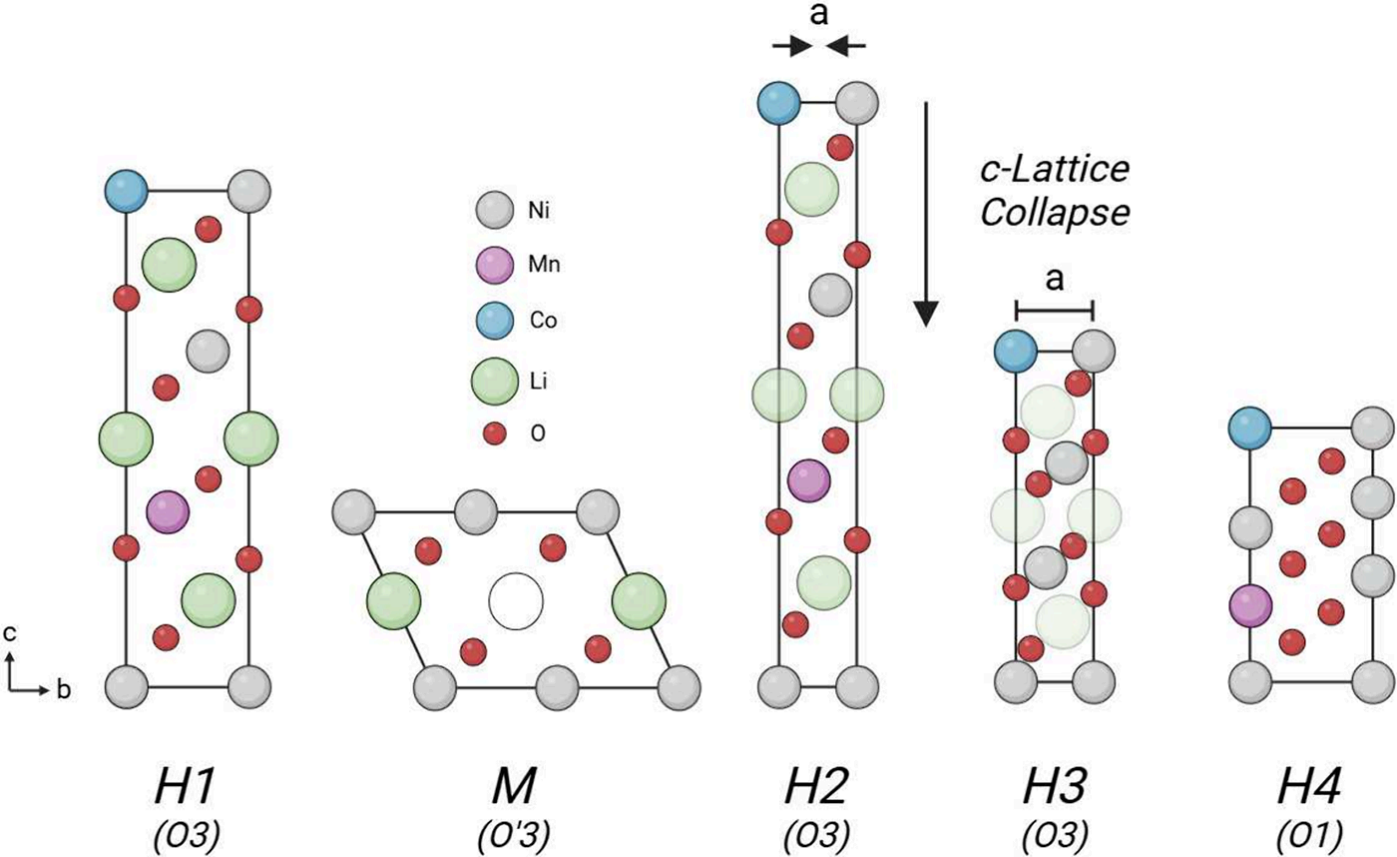
Diagram outlining the crystal structures associated with the O3-type H1, M, H2, and H3 phases, and the O1-type H4 phase. Differences in Li-ion occupancy are illustrated by increasing transparency, where H1 is the least delithiated and H4 is the most delithiated.

Between the terminal O3 and O1 phases, changes to the unit cell do still occur. These include the H1, M, H2, and H3 phase transitions (where H and M stand for hexagonal and monoclinic, respectively), whose associated structures are depicted in Figure 1. The formation of these phases can be identified through the measurement of crystallographic lattice parameters,^39^ where *a*, *b*, and *c* define the unit cell dimensions and α, β, γ define the angles between them. For the R3̅m space group, *a* = *b* ≠ *c* and α = β = 90°, γ = 120°. The *c*-lattice parameter defines the height of the unit cell along the stacking direction and includes contributions from both the Li and TM layers. While an expanded *c*-parameter is generally indicative of wider Li-ion diffusion channels, factors such as TM-layer spacing and stacking faults can also influence Li-ion mobility. Despite this complexity, a collapse in the *c*-parameter does often coincide with a drop in Li-ion kinetics,^24,40–44^ and helps to explain the poor transport properties of the H3 and O1 phases.

Illustrating the relationship between thermodynamic degrees of freedom and their influence on the electrochemical voltage profile, the Gibbs phase rule provides a strong foundational framework for interpreting the formation of the H1, M, H2, and H3 phases.^45^ For the two-phase transitions in LiTMO_2_, the participation of an additional electrochemically active phase reduces the thermodynamic degrees of freedom (DoF) to zero.^46^ The voltage becomes independent of the state of charge and a plateau arises in the electrochemical profile. This is accentuated in differential capacity (dQ/dV) profiles, where peaks indicate the onset, propagation, and reversibility of these phase transitions.^47^ Note that these are sometimes referred to as “redox peaks” in the literature.^48^ However, it is important to remember that redox (charge compensation) facilitates (de)­lithiation, which leads to structural changes that are observed in the electrochemical profile. The dQ/dV peaks are derivative features of that electrochemical profile and thus, depend on the number of available DoF;^24^ dQ/dV peaks are not caused by a particular redox reaction.

H1, M, H2, and H3 transitions have been observed in LiCoO_2_,^49–54^ and later LiNiO_2_,^55–59^ where two-phase reactions could be induced via chemical and electrochemical methods. Due to the volumetric expansion/contraction that accompanies these transitions, they are hypothesized to be a leading cause of particle cracking. Fortunately, the deleterious effects of these H1, M, H2, and H3 phase transitions in SC-NMC811 are limited by the disruption of long-range TM ordering with the random doping of Co and Mn onto Ni sites.

Nevertheless, the most severe crystallographic phase transition is the H2 to H3 transition. This is described by a significant contraction of the unit-cell height, marked by a decrease in the *c*-lattice parameter. In Ni-rich TM oxides, this phenomenon is commonly explained by invoking dynamic charge transfer from O to Ni beyond a critical state of charge.^42,60^ By increasing the formal charge on O, electrostatic interlayer repulsion is allegedly reduced, leading to a rapid contraction in the stacking direction. However, this explanation should scale with increasing Ni content and since the same phenomenon is observed in LiCoO_2,_ ^51,53^ additional considerations must be necessary.

Although Ni-rich NMCs and LiNiO_2_ primarily operate under the same redox couple, both systems exhibit different lattice parameter evolution. The discontinuous structural evolution of LiNiO_2_, versus the solid solution behavior of NMC, together with the knowledge that Ni­(III) redox centers are already in the negative charge transfer regime prior to the onset of *c*-lattice collapse,^25,32,61–63^ highlights that electronic effects are not responsible for the anisotropic structural changes. Instead, a recent study attributes lattice collapse to an increase in crystallographic strain that occurs beyond a critical state of charge.^64^ This explains why 80% delithiation results in the same shrinkage of the *c*-lattice parameters across a range of NMC chemistries.^42^ It also explains why SC particles of the same chemical composition do not crack, due to their better ability to withstand mechanical stress. For SC-cathodes, this has been modeled using a nonlinear Fickian diffusion model, where the results demonstrate that differences in Li-ion occupancy do not strongly couple to macroscopic stress evolution.^65^ This approach highlights the robustness of SC particles under operational conditions and reinforces the conclusion that mechanical degradation (cracking) in well-designed SC-NMC811 is primarily driven by external factors rather than intrinsic Li diffusion-induced stress.

### Ionic and Electronic Conduction

At the atomistic scale, Li-ion diffusion is a critical determinant of practical specific capacity. During (de)­lithiation, Li-ions migrate through face-sharing tetrahedra as they hop between octahedral 3a sites. In the O3 structure (Figure 2a), the ease with which this (de)­lithiation mechanism occurs partly depends on the Li-ion occupancy in the surrounding octahedra.^66^ If these adjacent sites are occupied, the Li-ions move into a tetrahedral site where Li–Li repulsion is strong (Figure 2b). However, if the adjacent octahedra are empty, this repulsion is absent, thereby providing a lower energy pathway for Li-ion conduction.^24,40^

**Figure 2:**
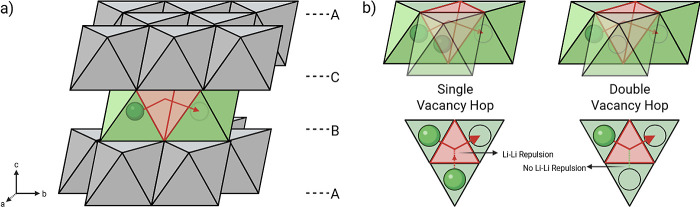
Illustrations of the O3 stacking and Li-ion diffusion pathway in a layered TM oxide Li-ion battery cathode. a) Polyhedral R3̅m crystallographic structure, where A, B, and C indicate the stacking order of the TMO_2_ layers. Li-ions are depicted as green spheres, with the tetrahedral intermediate site highlighted in red. b) Single and double vacancy hopping pathways are shown in side-on and top-down views, where a dashed red line indicates strong Li–Li repulsion, and a dashed green line indicates its absence.

LiNiO_2_ often exists as a series of off-stoichiometric, Li-deficient, Li_1–z_Ni_1+z_O_2_ (*z* < 1) phases, with an accurate crystallographic representation given by [Li^+^_1–z_ Ni^2+^_*z*_]_3b_[Ni^2+^_*z*_ Ni^3+^_1–z_]_3a_[O^2–^_2_]_6c_.^67^ This intrinsic cation disorder is commonly attributed to the comparable ionic radii of Li^+^ (0.76 Å) and Ni^2+^ (0.69 Å) in octahedral coordination,^68^ which promotes antisite defect formation through the partial substitution of Ni^2+^ onto Li^+^ sites. Considering the aforementioned ionic conduction pathway, the substitution of Li^+^ by Ni^2+^ at the 3*b* site (Li/Ni intermixing) obstructs Li-ion diffusion through the Li layer, thereby degrading the rate capability of the cathode material under study. The off-stoichiometry exhibited by these materials can be investigated through Rietveld analysis, wherein the isotropic atomic displacement parameter of Li (B_Li_) qualitatively indicates the excess electronic density arising from Ni^2+^ in the Li^+^ 3*b* site.^69^ Refinements can also be conducted to evaluate the degree of Li/Ni mixing, but not at the same time as the off-stoichiometry refinement since, to avoid overparametisation, a constraint must be applied that assumes the Li:Ni ratio is fixed at 1. To approximate the extent of Li/Ni mixing, it is also common to compare the integrated intensities of the 003 and 104 peaks.^57^

Alongside ionic conductivity, electronic conductivity is also important to consider. In the bulk structure, as Li-ions move in one direction toward the anode, electrons flow in the opposite direction toward the current collector. Due to the charge of both species, localized electrostatic interactions can deform the surrounding local environments. These distortions are described as quasi-particles called polarons, which represent the coupling between a charge carrier and its self-induced local distortion of the surrounding crystal structure.^70^ In LiTMO_2_, the structural distortion is limited to the first coordination shell.^71–73^ As such, these quasi-particles are termed “small polarons” (Figure 3a), which migrate via a thermally activated hopping process (Figure 3b).^74^ Although localized, polaron transport can significantly influence the conductivity and kinetics of the system.^75,76^ In extreme cases, a sluggish polaron can impede both ionic and electronic conduction. This is seen in other systems like LiMnPO_4_ and V_2_O_5_.^77,78^ Fortunately, this is not the case for Co- and Ni-containing LiTMO_2_ cathodes. However, understanding polaron transport does help elucidate trends in electronic conductivity. Polaron formation energy is lower in LiNiO_2_ than LiCoO_2_, thus, electrical conductivity increases as Ni content rises throughout the NMC series.^75,79,80^

**Figure 3:**
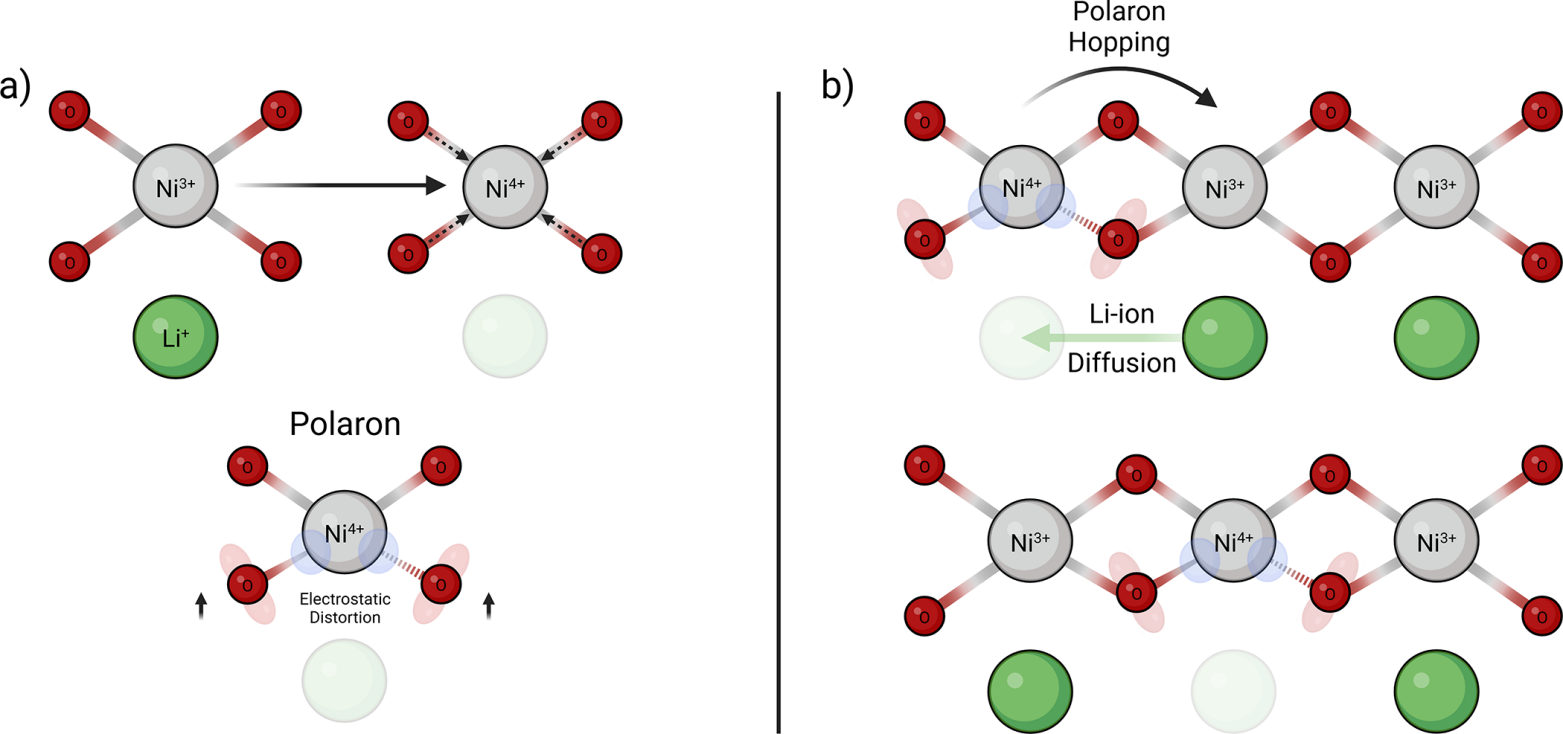
Diagrams depicting small polaron formation and migration in a Ni-rich layered TM oxide. a) Polaron formation caused by electrostatic repulsion from the net negatively charged Li vacancy. Wedged and dashed bonds indicate out-of-plane and behind-the-plane bonding, respectively, illustrating the puckering of the octahedral coordination environment. b) Migration of the small polaron opposite to the direction of Li-ion diffusion, outlining their intertwined transport mechanisms.

### Kinetic Limitations and Li-Ion Heterogeneity

Kinetic limitations, be they ionic or electronic, can lead to Li-ion heterogeneity, but experimentally determining the length scale at which this occurs in SC and PC cathodes is challenging.^64,81,82^ Addressing this, new techniques, such as interferometric scattering microscopy (iSCAT) (Figure 4a and 4b), can be used to study this behavior in monolithic SC particles. A seminal study employing iSCAT demonstrated that Li-ion heterogeneity emerges during charging, with a Li-poor periphery and Li-rich core forming due to concentration-dependent diffusivity (Figure 4c).^83^ During discharge, kinetic limitations at near-full lithiation result in incomplete Li-ion reinsertion into the core of SC primary particles, thereby contributing to poor first-cycle Coulombic efficiency. This heterogeneity persists across a broad spectrum of charging rates, ranging from C/30 to 2C. Notably, larger particles demonstrate significantly greater heterogeneity, which intensifies internal stress, impairs Li-ion kinetics, and accelerates electrochemical degradation.

**Figure 4:**
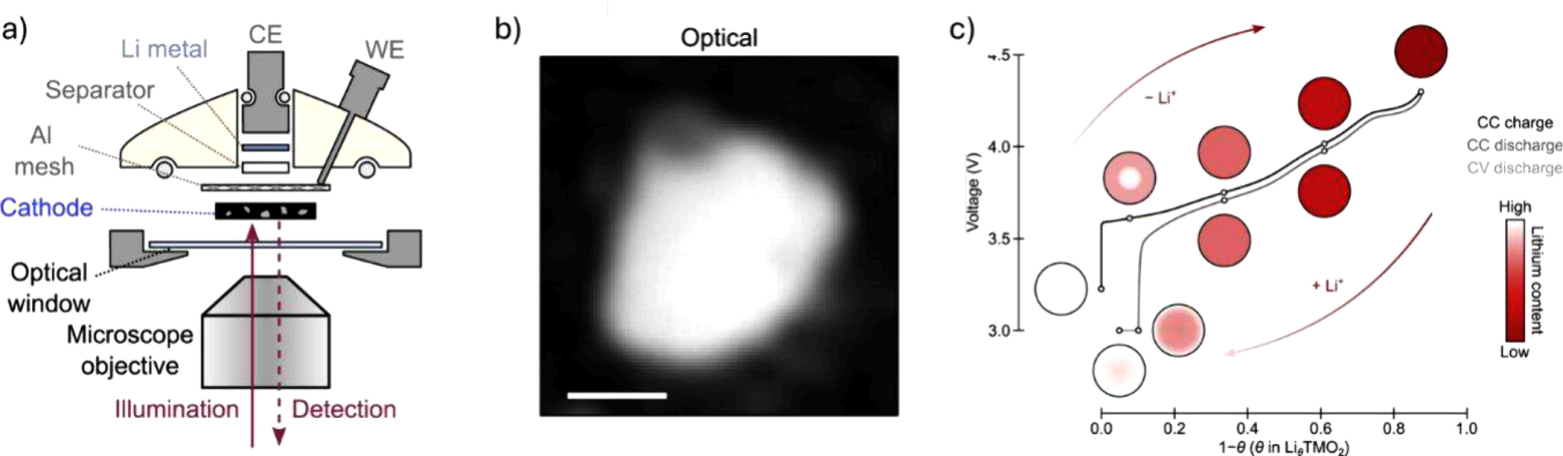
a) Schematic drawing of the key components of the electrochemical cell for optical microscopy. The cathode is a self-standing electrode composed of numerous NMC particles, carbon black and polytetrafluoroethylene (PTFE) binder. Aluminum mesh is used as a current collector (WE, working electrode; CE, counter electrode). b) Optical image of an active NMC particle in the electrochemical cell. Scale bar = 1 μm. c) Summary of the Li-ion distribution within the single active particle at various Li content. The circles show schematic representations of single-particle Ni-rich materials at various states of charge (SoC). The voltage profile is illustrative of the single-crystal NMC material used in this work and was obtained in a half-cell cycled with a CC charge and CC discharge (C/20 rate) and a discharge CV hold at 3 V (for 24 h). Reproduced with permission from ref 83. Copyright 2022 Elsevier. Licensed under CC-BY 4.0.

While *c*-lattice collapse is a well-studied phenomenon in SC or PC morphologies, and its association with particle cracking is well established, cracking is rarely the primary cause of performance fade.^29,30,84,85^ This is supported by ref 84, which demonstrates that particle cracking in PC particles can be observed as low as 3.8 V vs Li^+^/Li, with comparatively little occurring between 4.2 and 4.4 V (where the majority of degradation occurs).^29^ Furthermore, another study that used X-ray computed tomography showed that cracking was not observed in SC-NMC811 cathodes that were cycled under harsh conditions (2.5–4.4 V and 40 °C for 100 cycles at a C/3 rate).^39^ As such, the disproportionate capacity fade observed in SC or PC Ni-rich cathodes cycled up to 4.4 V cannot be attributed to particle cracking. Instead, capacity fade should be attributed to kinetic effects,^86^ where interfacial stability, Li-ion heterogeneity, and parasitic surface reactions play a more significant role in determining the practical capacity. Herein, lies the greatest advantage of SC-cathodes.

### Morphological Differences

The primary difference between SC and PC morphologies lies in the evolution of their respective surface areas during electrochemical cycling. The surface areas of pristine SC and PC particles do not exhibit a clear trend since they are largely determined by the synthesis method. However, consistent differences do emerge in how these surface areas evolve during electrochemical cycling. These trends are typically investigated using gas adsorption data and the Brunauer–Emmett–Teller (BET) equation.

For materials with low surface areas, like LiTMO_2_ primary and secondary particles, Kr gas adsorption at 77 K is the IUPAC recommended gas adsorption method due to its superior sensitivity.^88,89^ This not only provides surface area but also valuable information on pore volume and pore size distribution,^90^ enabling a detailed analysis of material structural evolution so long as measurements are conducted within the appropriate BET interval (p/p_0_ = 0.05–0.3). One study utilizing Kr physisorption showed that for SC-NMC811 cathodes, only a slight decrease in gas adsorption and BET surface area occurred after charging to 4.2 V versus Li^+^/Li.^87^ This result aligns with the known volumetric contraction of SC-NMC811 during delithiation,^91^ together with the lack of mechanical fracturing that is afforded by the stable SC morphology. In stark contrast, the BET surface area of the corresponding PC-NMC811 cathode increased (from 0.2 m^2^ g^–1^ to 1.4 m^2^ g^–1^) when charged to the same voltage. This observation suggests that particle cracking plays a leading role in driving the increased surface area of PC-NMC811. While both cathodes consist of the same chemical composition and presumably benefit from the same degree of disruption to the long-range TM ordering, the grain boundaries present in PC-NMC811 appear to act as nucleation points for this mechanical stress to manifest and grow into a crack.^92^ Although this increase in surface area may seem beneficial for ionic diffusion by offering a shorter path length and greater electrolyte coverage it also introduces challenges. The presence of reactive surface sites, which are prevalent in highly oxidized Ni-rich TM oxides,^26^ promotes parasitic reactions.^93^ These reactions can negatively impact long-term electrochemical performance, as will be discussed in a subsequent section.

## Single Crystal Synthesis

To understand the synthesis pathways of SC cathodes, it is essential to first outline the methods by which current-generation cathodes are synthesized. In general, LiTMO_2_ cathodes are synthesized through combination of a TM precursor (pCAM), typically a hydroxide or oxide, with a Li source such as Li_2_CO_3_ or LiOH. The mixture is then subjected to high-temperature calcination under air or O_2_ (depending on the Ni content), which allows for the formation of the desired R3̅m layered LiTMO_2_ phase/cathode active material (CAM). For a PC cathode, morphological control is achieved during the creation of the TM precursor. These cathodes utilize TM­(OH)_2_ spherical secondary particles (with diameters in the region of 5–20 μm). The spherical morphology offers the lowest surface area-to-volume ratio, effectively minimizing any undesirable surface reactions between the electrode and the electrolyte. However, repeated anisotropic expansion and contraction can instigate particle cracking, where microcracks are generated at both the primary and secondary particle level.^94–99^ This cracking behavior exposes fresh surfaces for participation in parasitic reactions and is proliferated on continued cycling. As a means to ameliorate this, SC cathodes, a collection of monolithic primary particles with diameters of 1–5 μm (Figure 5), can be used. By adopting this grain-boundary-free morphology, intergranular cracking can be circumvented, thereby mitigating surface degradation and presenting a promising approach in improving the cycle lifetime of Ni-rich layered oxide cathodes.

**Figure 5:**
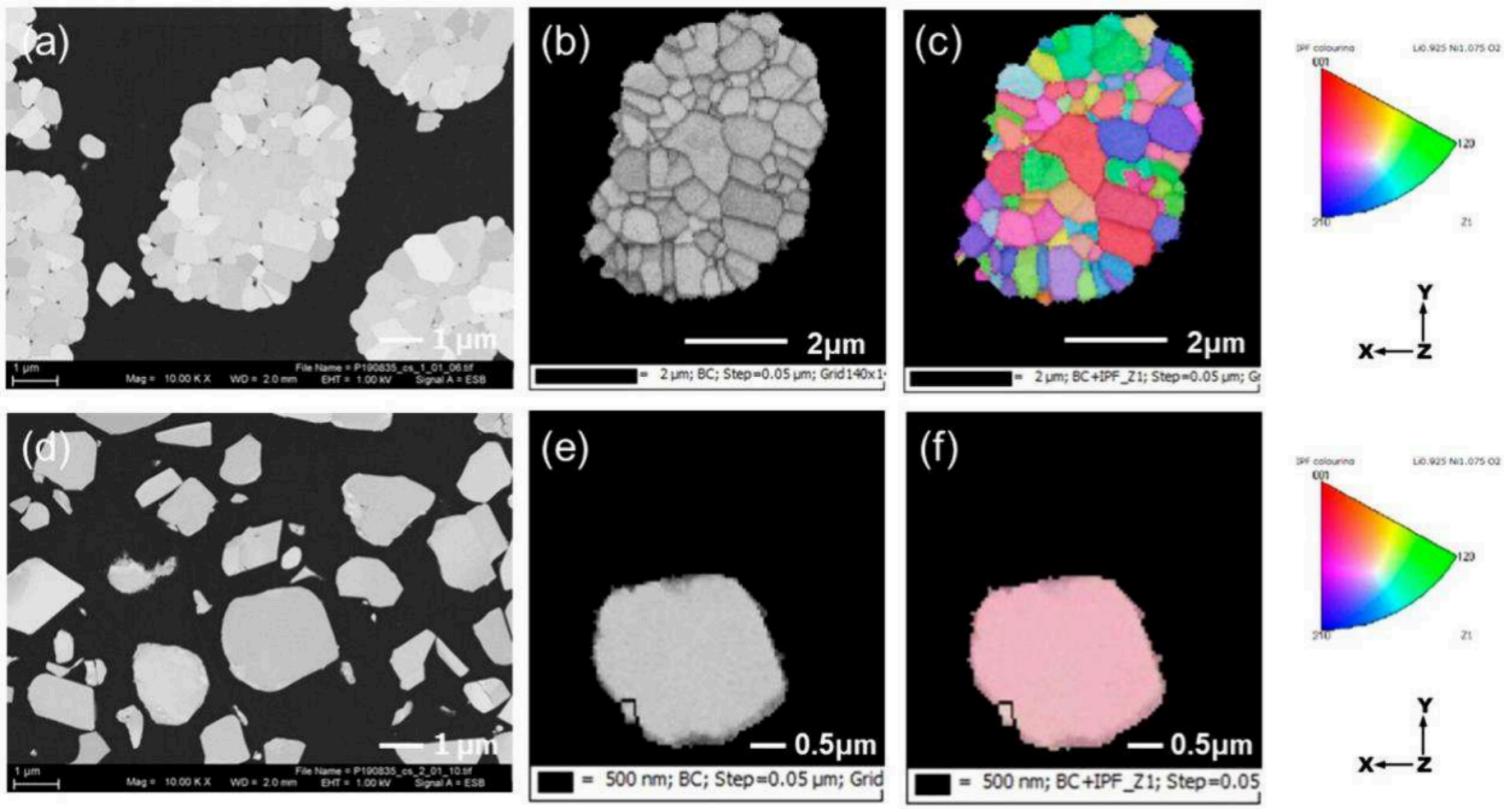
Comparison of SC and PC morphologies and the crystallographic periodicity observed throughout their respective bulk structures. Cross-sectional SEM images and EBSD mapping of (a–c) PC-LNO and (d–f) SC-LNO. (a, d) Backscattered electron images, (b, e) band contrast maps, and (c, f) crystal orientation maps of the particles shown respectively in b and e. Reproduced with permission from ref 102. Copyright 2022 American Chemical Society.

The definition of SC in its use to describe the morphology of Li-ion battery cathode materials remains somewhat ambiguous and, in the literature, there remains some disagreement as to what constitutes a “single crystal” material. The authors appreciate the subjectivity of this term and to avoid confusion, in this review we will adopt the convention of Mesnier and Manthiram in defining a SC morphology.^100^ First, particles should be as deagglomerated as possible, and any remaining agglomerates should comprise of less than 5 crystals. Second, to differentiate between SC cathode particles and nanoparticles (which display poorer tap densities and higher surface reactivities), the SC particles should possess an average particle size greater than 1 μm.^101^

The synthesis of high-quality SC Ni-rich layered oxide cathodes presents a complex challenge. For PC materials, synthetic approaches are well understood and reasonably standardized throughout the literature, whereas the SC synthesis of Ni-rich cathodes remains a nascent field. In theory, the larger individual primary particles that define the SC morphology can be easily grown under high temperature annealing conditions during lithiation. This is because of the exponential dependence of particle growth with temperature.^101,103^ However, the intense conditions required for SC growth increase the difficulty of producing high-quality Ni-rich materials. Regardless of morphology, synthesis at excessively high temperatures leads to structural degradation, exacerbating cationic disorder, and promoting the formation of rock-salt phases. These issues collectively increase electrochemical impedance and diminish cycling performance.^104,105^ As such, this section will discuss the variety of approaches reported in the literature for synthesizing SC Ni-rich layered oxide cathodes, scoping from traditional routes that mirror PC synthesis, to more complex methods that stimulate particle growth without excessively high temperatures. How synthetic conditions affect particle size, and the emergence of dominant crystal facets will also be discussed. Finally, scale-up considerations for the synthetic routes will be examined to assess the viability of these pathways for commercial realization.

### Quality Markers for Ni-Rich Layered Oxide Cathodes

CAMs are ultimately assessed on their electrochemical performance - how well they can satisfy the desired metrics of energy density, power, lifetime and safety. As before, electrochemical properties are influenced by the structure and morphology observed at the atomic- and particle-level. Since the synthetic conditions ultimately govern these properties, one can appreciate the importance of cathode synthesis in delivering high-quality CAMs.

For SC Ni-rich layered oxide materials, the quality can be assessed through a combination of physical properties: the chemical composition, degree of Li/Ni mixing,^106–109^ degree of crystallinity and/or impurities,^110,111^ particle size,^112,113^ and tap density.^114^ These physical properties are governed by the synthesis parameters (e.g., precursor choice, calcination temperature and calcination atmosphere) with material quality becoming particularly sensitive to reaction conditions as the Ni-content increases. Application of excessive temperatures in SC-LiNiO_2_ materials led to Li^+^/Ni^2+^ mixing (from approximately 2 to 5% for temperatures of 675 and 800 °C respectively) and subsequently lower deliverable capacities during cycling (decreasing from 205 mAh g^–1^ to 170 mAh g^–1^ as the temperature was increased). While this section will primarily focus on discussing the various synthetic pathways that have been used to achieve SC morphologies with a Ni-rich stoichiometry, the physical properties and resulting electrochemical performance will also be referenced.

### Synthetic Pathways

#### Co-Precipitation

The most popular route to Ni-rich layered oxide cathode active materials is undoubtably hydroxide coprecipitation, wherein TM­(OH)_2_ pCAM powder is precipitated from a solution containing (i) dissolved TM salts, (ii) a precipitating agent, and (iii) an alkaline complexing agent.^115^ These are most commonly (i) TM sulfates, (ii) NaOH, and (iii) aqueous ammonia, respectively, with reactions typically carried out in a stirred tank reactor (batch) or a Taylor vortex reactor (continuous). The complexing agent and tightly controlled reaction conditions (pH, stirring speed, temperature, addition, and residence times etc.) yield the spherical TM­(OH)_2_ secondary particles.^116–119^ Fortunately, the spherical nature of the as-synthesized particles is retained during the lithiation step and leads to the production of the familiar “meatball” shape. Although this synthetic route is primarily associated with the production of PC cathode materials, the atomic-level mixing it provides means that coprecipitated precursors are also widely utilized in the production of SC CAMs. In this case, the SC morphology arises from additional milling steps conducted before or after calcination, as well as adjustments to the lithiation conditions that deviate from those that are typically used to preserve the PC morphology. For example, by raising the calcination temperature from 750 to 850 °C (and employing a postcalcination milling step for the SC sample), SC- and PC-LiNi_0.9_Co_0.05_Mn_0.05_O_2_ could be synthesized, with both materials displaying similar levels of antisite mixing (≈ 2.3%) and similar lattice parameters.^120^ Here, despite similar (and low) antisite mixing levels, both lower initial discharge capacities and more severe capacity fade was seen in the SC CAM compared to PC, demonstrating the challenges in synthesizing high performance, SC-Ni-rich CAMs. These trends appear reversed in the reported synthesis, where both SC- and PC-LiNi_0.8_Mn_0.2_O_2_ materials were produced from Ni_0.8_Mn_0.2_(OH)_2_ at a calcination temperature of 780 °C, with SC morphologies achieved through initial jet milling of the hydroxide precursor followed by a postcalcination annealing step at 500 °C.^121^

Hydroxide pCAM with different sizes is also reported to influence the final CAM morphology.^122^ In this context, Zeng et al. reported that 3–5 μm sized precursors were used to produce SC NMC811, while larger 10–12 μm sized precursors yielded PC NMC811.^122^ Notably, the calcination temperature was only increased from 760 to 800 °C between the two final products, with similar antisite mixing (3.3% vs 3.9% for SC and PC respectively), initial discharge capacities (approximately 200 mAh g^–1^ at 0.1C) and rate capabilities (up to 5C) observed between the two CAM morphologies. After 100 cycles at 1C, the SC CAM was capable of retaining 86.7% of its capacity compared to 73.8% for the PC CAM when cycling to 4.3 V vs Li+/Li (80.1 and 50.6% respectively when the upper cutoff voltage was increased to 4.5 V vs Li^+^/Li) demonstrating the superior capacity retention of the SC CAM here. The as-produced SC-NMC811 particles were 2–4 μm in size, matching the size of the secondary precursor particles. This suggests that the SC particles form through fusing of the primary precursor grains at high temperature.

Much of the work reported by Dahn’s group on SC-Ni-rich layered oxide cathode synthesis uses spherical hydroxide pCAM with some approaches employing a two-step lithiation procedure to decouple the particle growth stage from the phase formation stage.^123–127^ In SC-NCA, prepared through a one-step high temperature route from hydroxide pCAM, a Li_5_AlO_4_ impurity is formed due to quick Al segregation at high temperatures.^128^ By employing a two-step lithiation method, this impurity could be eliminated.^129^ First, an Li-poor material is formed (Li/TM between 0.8–0.975) at temperatures between 850–900 °C, a range which promotes SC particle growth. The second lithiation step, performed at 735 °C, brings the Li/TM ratio up to 1.02, significantly decreases the Li^+^/Ni^2+^ mixing (from ≈ 9% to <1% in the most extreme case presented in this study) and produces the final cathode material well-preserved particle sizes (likely due to the limited particle growth that would occur at the lower temperature), indicating the effectiveness of this additional lithiation step. Compared to a PC CAM of the same composition synthesized at 735 °C, slightly higher antisite mixing was calculated and when cycled, display nominally lower capacities, similar capacity retention and higher kinetic hindrance for the SC morphology. Doped LNO materials (Al, Mg) have also been formed through the two-step lithiation method.^130^ At temperatures (for the first heating step) increasing from 850 to 950 °C, the Li/Ni mixing is increased in a roughly linear fashion for most iterations presented in the study (from ≈ 4 to 12% in the most extreme case). However, the Li_5_AlO_4_ impurity is still formed, and the SC materials here display no electrochemical improvements when compared to an analogous PC sample of the same chemical composition, emphasizing the difficulties encountered in synthesis approaching ultrahigh Ni compositions. Doped LNO materials (Al, Mg) have also been formed through the two-step lithiation method.^130^ However, the Li_5_AlO_4_ impurity is still formed, emphasizing the difficulties encountered in synthesis approaching ultrahigh Ni compositions.

While the synthesis of SC Ni-rich cathodes is still dominated by spherical hydroxide pCAM materials,^131^ it is worth questioning how necessary these are as starting materials. While in academic research these materials are widely available and are presumably used out of habit, for commercial manufacture, cutting out the coprecipitation step has the potential for large cost and energy savings.

#### Solid State

Solid-state synthesis is considered to be the traditional “shake and bake” route to forming layered oxide materials, where individual TM and Li salt precursors (often oxides or carbonates) are mechanically mixed prior to high temperature heat treatment, often with several intermediate grinding steps to improve homogeneity in the final sample. Though similar, this differs from the coprecipitation method where the mixed-metal pCAM with defined morphology and atomic-scale mixing of TMs, is produced through a solvent-heavy and cost intensive process.^132–134^ From an economical perspective, solid-state routes could be viewed as advantageous owing to their solvent lean nature, high yield, and lower number of processing steps.^135^ For ternary metal oxide type materials, LiTMO_2_, higher temperatures, longer calcination times, and pelletization are required to promote the solid-state diffusion that converts the precursor salts to the CAM product.

Historically, solid-state methods were widely used in layered oxide cathode synthesis. For example, the LiCoO_2_ cathode material in Mizushima and Goodenough’s seminal 1981 paper was produced through heating a pelletized mixture of Li_2_CO_3_ and CoCO_3_ in air at 900 °C for a total of 60 h.^13^ Similar methods have been used for Ni-rich materials whereby LiNiO_2_ and LiNi_1–*y*_Co_*y*_O_2_ (*y* = 0.1, 0.2, 0.3, 0.5) were obtained by direct reaction of NiO, Co_3_O_4_, and Li_2_CO_3_ at calcination temperatures between 800 to 850 °C for up to 40 h.^136,137^ Although the particle morphologies for LCO, LNO and LiNi_0.8_Co_0.2_O_2_ are not shown here, it is reasonable to suppose that the chosen precursors paired with the reaction conditions give rise to large SC-type particles; the hierarchical morphological control required to produce spherical PC agglomerates is typically carried out prior to the calcination step.

While hydroxide coprecipitation has dominated cathode materials synthesis, there has been renewed interest in dry solid-state methods to eliminate cost and energy intensive manufacturing. For example, Zheng et al. have explored the synthesis of SC LiNi_0.6_Mn_0.2_Co_0.2_O_2_ (NMC622) through an all-dry method.^138^ Here, binary metal oxides were milled to form a submicron size precursor where a homogeneous, solid-solution phase of these precursors was achieved by ball-milling for 1 week, while high-energy ball milling for 1 h yielded a mix of nanograined NiO, MnO and Co_3_O_4,_ indicating poor mixing of the transition metals. However, it was found that here that atomic-level mixing was not a prerequisite, where SC-NMC622 particles of 3–10 μm in size could be grown at 940 °C with a calcination time of only 12 h. This study reported that repeating the process under O2 restricted particle growth and yielded significantly smaller particles (between 1–2 μm) compared to heating in air. Additionally, decreased Li/Ni mixing (from >3.5% for samples calcined in air to <2% for those calcined under O2) and improved first-cycle Coulombic efficiencies (≈85% compared to ≈80% for air-calcined samples) were achieved, demonstrating the efficacy of O2-rich calcination environments for Ni-rich materials. This has significant implications for promoting particle growth in SC-Ni-rich materials, where heating steps must be conducted under O_2_ to maintain low antisite mixing levels and mitigate against O vacancy formation.^139,140^ To combat this effect but still promote sufficient particle growth, a two-step heating procedure was implemented–initially exploiting good particle growth under air followed by heating under O_2_ to mitigate against the aforementioned structural defects. Antisite mixing levels in this case were still kept below 2% and the resulting electrochemistry was improved, showing a ≈87% first cycle Coulombic efficiency and ≈ 90% capacity retention after 90 cycles, although it is noted that, comparing this to an earlier study,^124^ where the same group prepared SC-NMC622 from a coprecipitated hydroxide precursor, rate capabilities are inferior for the all-dry method. Moving toward increased Ni-content, Ruess et al. report the synthesis of SC-LiNi_0.83_Co_0.11_Mn_0.06_O_2_ with reduced calcination and annealing times without the need for pelletizing or ball milling.^135^ Here 2–3 μm SC were produced by blending NiO, MnO, and Co_3_O_4_ for just 1 min. The blended powders then underwent calcination between 875 °C–975 °C for 9 h followed by pre- and postwashing annealing steps at 750 °C for 3 h. Interestingly, antisite mixing was decreased from ≈8% to ≈ 3% by increasing reaction temperature, however, this is rationalized by the synthesis at 875 °C not being sufficient to overcome kinetic limitations. By using binary metal oxide precursors instead of a mixed metal hydroxide, the authors concluded that the reaction becomes controlled by kinetics rather than by thermodynamics (in the hydroxide case), requiring high temperatures and long reaction times. The synthetic route adopted by the authors here provides an energy saving up to 30% (66 MJ kg^–1^) as compared to hydroxide coprecipitation. This immediately presents an economic benefit if “shake and bake” methods for SC synthesis were to be revisited.

#### Alternative Solution-Based Routes

While the coprecipitation and “shake and bake” routes represent the majority of synthesis procedures reported for SC Ni-rich layered oxide materials, other techniques including hydrothermal, spray pyrolysis, and sol–gel are reported in the literature.^141–147^ In these, and as within coprecipitation, each technique aims to produce a precursor material with good atomic-level mixing of TMs in an effort to overcome kinetic barriers during synthesis. These solvent heavy techniques force comparison to the poor economics of the coprecipitation reaction. However, they may provide interesting routes toward bespoke particle morphologies and access of different crystal facets which can alter the surface properties of the resultant cathode material. Rod-like particle morphologies of NMC811 are possible through hydrothermal routes, where a rod-shaped oxalate precursor was produced hydrothermally in an autoclave at 180 °C for 2 h from a solution of Ni and Co acetates mixed with urea as a shaping agent. This precursor was further mixed with LiOH.H_2_O and Mn­(NO_3_) and subject to calcination at 750 °C for 12 h under O2, where the rod-like morphology was broadly retained.^148^ Compared to PC-NMC811, the rod-like SC-NMC811 here (calcined with a 50 mol % excess of Li) showed superior cycling stability in half coin-cells over 100 cycles. However, out of the six SC-NMC811 variants reported here (with Li excess amounts from 10 to 60 mol %), only one showed improved performance, with the 50% excess Li adding increased cost to the process. Lu et al. reported a hydrothermal assisted route to obtain rare polyhedron shaped SC-NMC811 materials, displaying predominantly (104)-type facets.^149^ For this synthesis, TM acetates and urea were dissolved in an ethanol/water mixture in a Teflon lined autoclave to yield a Ni_0.9_Co_0.1_Mn_0.1_CO_3_ precursor on heating between 160–200 °C for 24 h. Both this carbonate precursor and a traditional hydroxide precursor (from coprecipitation) were mixed with LiOH.H_2_O and calcined between 900–930 °C for 20 h, with resulting materials displaying polyhedral and octahedral facets, respectively. This highlights the ability of the precursor to determine the final SC morphology and observed facet composition. Electrochemically it is shown that the polyhedron SC-NMC811 here showed higher initial discharge capacities, better rate capability and improved capacity retention over 100 cycles at both 1C and 6C compared to octahedron shaped SC-NMC811 and conventional polycrystalline NMC811.^149^ Superior surface stability shown through Ni L-edge XAS, HRTEM imaging and gas evolution studies (where minimal CO_2_ and O_2_ evolved compared to polycrystalline CAMs) was also observed for polyhedron shaped SC-NMC811 here, demonstrating the benefits of targeting specific, more stable facets.

All of the above approaches rely on the growth of SC cathode particles through solid-state grain boundary particle growth and resultantly, insights from one route may inform others. Although this mechanism is effective at high temperatures and over long reaction times, the thermodynamic instability of Ni-rich (>90%) layered oxides imposes difficulties. For the most Ni-rich, LNO, structural degradation begins to occur at temperatures as low as 700 °C.^104^

#### Molten Salt Approach

To induce particle growth at lower temperatures and thus overcome the temperature sensitivity problem in Ni-rich layered oxides, molten-salt synthesis is a promising route. As the name suggests, salts with a melting point far below the desired synthesis temperature are used to provide a liquid medium/flux through which particle growth can occur through a dissolution-recrystallization mechanism (Figure 6). This affords greater mass transport compared to solid mediums, prevents agglomeration, and promotes the growth of uniform SC particles.^101^ These favorable conditions allow for SC growth to occur at the same temperature as for PC-NMC synthesis, thus helping to mitigate against temperature induced structural degradation. A wide variety of Li and non-Li constituent salts have been reported, including NaCl, KCl, CsCl, LiCl, Li_2_SO_4_, Na_2_CO_3_, LiNiO_3_, H_3_BO_3_, and any resulting mixtures thereof.^150–153^ Evidently, these salts span a wide compositional range, and as long as melting point of any mixture is far below the annealing and calcination temperatures, successful molten salt synthesis can occur. As a slight drawback, this route does necessitate postcalcination washing steps for removal of the salt species, which then necessitates a postwashing annealing step to heal any surface damage caused by water exposure. The inclusion of additional salts in the flux also adds extra cost, although this may be mitigated by recovery, recycling, and reuse of the salts obtained from previous postcalcination washing steps.

**Figure 6:**
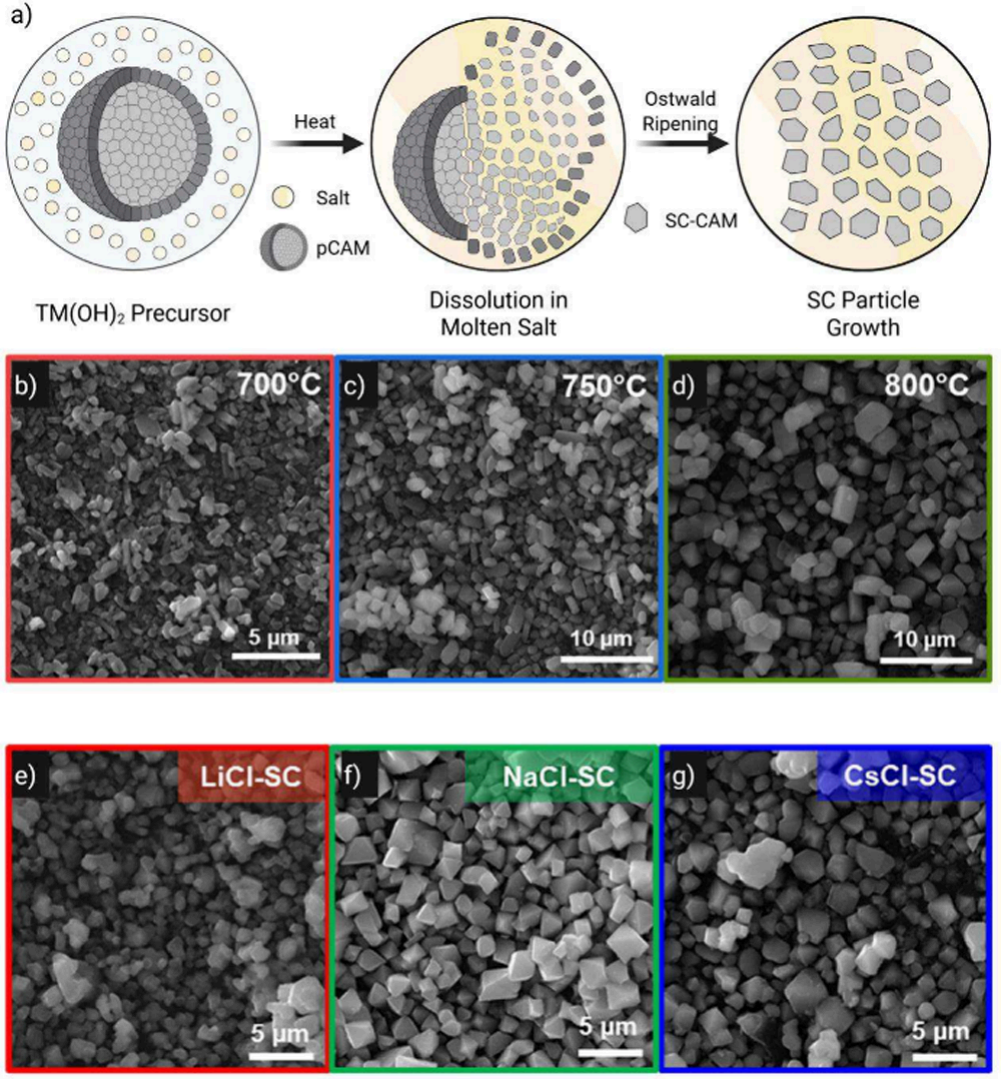
a) Schematic of pCAM dissolution and SC-CAM growth in a molten salt flux. (b–d) SEM micrographs of SC-CAM synthesized at 700 °C (red), 750 °C (green), and 800 °C (blue); (e–g) SEM micrographs of SC-CAM synthesized using LiCl (red), NaCl (green), and CsCl (blue) fluxes. Panels (b-g) reproduced with permission from ref 150. Copyright 2023 American Chemical Society.

The synthesis of SC-NMC811 has been reported as far back as 2012 by Kim, who assessed the role of KCl and NaCl fluxes in the material properties.^151^ Starting with a spherical Ni_0.8_Co_0.1_Mn_0.1_(OH)_2_ precursor, primary particle agglomeration still occurred in samples calcined at 800 °C despite the use of a flux. However, SC morphologies were shown to emerge at 900 and 1000 °C, with the NaCl flux yielding larger particles at a given calcination temperature when compared to the KCl flux. Furthermore, while the KCl flux promoted the growth of isotropic particles with a range of crystal facets, octahedral shaped particles were grown using NaCl and consisted of (0 0 3), (1 0 1), and (1 −1 −1) equivalent facet planes. The XRD data show low (0 0 3)/(1 0 4) intensity ratios for the SC-NMC811 samples here, indicative of increased Li/Ni disorder most likely due to the high temperatures (900 °C, 1000 °C) used. Coin half-cell data showed that initial capacities were comparable to those achievable by NCM532 (≈ 180 mAh g^–1^ at 4.45 V vs Li^+^/Li) and lower than would be expected for NMC811 likely arising from antisite mixing. As described previously, the flux can contain Li salts which can act as the liquid reaction medium and also as the Li source. For example, the molten salt route to SC-NMC811 adopted by Lüther et al. utilized a LiOH/Li_2_SO_4_ flux (melting point <500 °C) with a LiOH/TM molar ratio of 1.5 and a Li_2_SO_4_/TM molar ratio of 0.25 (note that LiOH:TM ratios for PC counterparts typically range from 1.01–1.07).^154^ This provided enough Li to maintain the efficacy of the flux throughout the reaction while also facilitating the lithiation of the Ni_0.8_Mn_0.1_Co_0.1_(OH)_2_ precursor. Calcination was carried out at temperatures of 800, 850, and 900 °C to obtain differently sized SC particles ranging from ≈ 2–5 μm with size increasing linearly with temperature. The degree of Li/Ni antisite mixing is lower across all three SC-NMC811 materials compared to PC-NMC811 calcined at 800 °C with no flux, suggesting that enhanced ion mobility allows for a more controlled crystallization process, although this defect concentration is tripled from 0.3% to 0.9% on increasing the SC-NMC811 reaction temperature from 850 to 900 °C. However, lower initial discharge capacities are observed for SC-NMC811 CAMs here although improved long-term capacity and voltage retention is observed in graphite cells.

Clearly, the infinite number of possible flux compositions yield a large space for optimization. Therefore, understanding the role of the salts and their effect on the resulting morphology is key in informing design strategies for high performance SC-NMC cathode materials. In a study to guide design molten-salt strategies for Ni-rich SC cathode materials, Mesnier and Manthiram investigated a total of 16 different flux mixtures varying in salt identity and composition.^100^ Two key issues were highlighted. First the chemical potential of O in the molten salt is low and acts as a roadblock to sufficient oxidation of the TMs. To fix this, salts also considered oxidizing agents such as Li_2_O_2_ and/or LiNO_3_ can be incorporated into the flux, where the addition of Li_2_O_2_ in addition to LiOH reduced the Li/Ni cation mixing from 18 to 11% (although this is still excessively high in reality). Second, nonparticipating fluxes (i.e., non-Li-containing salts) may be beneficial in producing homogeneous particles. While Li-containing fluxes reduce complexity regarding salt choice, they may sublime/evaporate during calcination due to their high volatility and hinder the dissolution-recrystallization mediated growth, resulting in broad particle size distributions. Taking these factors into account, the authors demonstrated that SC-LiNiO_2_ from a NaCl, LiOH, Li_2_O_2_ and LiNO_3_ quaternary flux was capable of outperforming PC-LiNiO_2_. With this specific flux composition, the cation mixing could be reduced to just 1.84% (compared to 1.91% calculated for the PC-NMC811 used in this study) at a calcination temperature of 675 °C (increasing to 2.14% and 4.32% as the temperature increased to 750 and 800 °C). It is important to note that only one of 16 different flux compositions could produce a SC cathode with superior electrochemical performance over its PC counterpart, and that this performance showed improvements only in capacity retention and not specific capacity. This reaffirms the authors’ original statement that understanding the mechanism and role of the flux is important and remains a promising avenue for future study.

### Particle Size Considerations

Controlling particle size during SC synthesis is critical as it can greatly impact the electrochemical performance. Calcination temperature and choice of flux (for molten-salt synthesis) have the largest impact.^155^ For example, in the molten-salt synthesis of SC-LiNiO_2_ a LiOH:Li_2_O_2_:LiNO_3_ flux at calcination temperatures of 650, 750, and 800 °C yielded average particle sizes of 400 nm, 1 um and 3 μm, respectively. Although even smaller particles <250 nm were also present,^100^ high Li/Ni mixing and low capacities were apparent in the 800 °C sample. By adding NaCl, the number of sub-250 nm particles were reduced, thereby highlighting the benefits of including at least one non-Li containing salt in the flux. Although controlling the particle size can be achieved with a judicious choice of reaction conditions, the effect of particle size on cycling behavior must be understood. Generally, SC particles display poorer kinetics for Li-ion transport when compared to their PC analogue and therefore exhibit worse rate capability during cycling. This limits their use in high current rate applications.^124,156^ In the generalized case, the larger path lengths in micron-sized SC particles increase the diffusion time according to Fick’s second law.^157–161^ Simply reducing the size of the SC particles to submicron level will improve these kinetics but will exacerbate aggressive surface reactions to the detriment of electrochemical stability. Therefore, a balance must be struck regarding size and surface area.

### Surface Facet Optimization

While the particle size of SC materials can be controlled through synthesis, the shape can also be tailored. The formation of specific surface facets during synthesis gives rise to particles with distinct geometries.^162,163^ For LiTMO_2_ layered oxides, SC particles are enclosed by (012), (001) and/or (104) facets whose surface fraction be controlled by the chemical potential of O and Li during synthesis.^164^ Experimentally, particles are likely enclosed by all three facets in varying surface fractions due to the difficulties in practically achieving particles dominated by only one or two facets. It has been shown through DFT that the (012) and (104) facets are the least and most stable, respectively Unfortunately, the (012) facet also exhibits the greatest Li-ion diffusivity. Zhu and Chen have demonstrated the molten-salt synthesis of SC-NMC333, 532, 622, and 811 with faceted morphologies with octahedral, truncated-octahedral, polyhedral and plate shapes produced through a molten-salt route.^165^ Here, precursor choice, flux to TM ratio, time, and temperature all influenced the resulting morphology and emergence of faceted surfaces. On moving to higher Ni-content materials (Ni content >90%), controlling the O_2_ environment during heating should push the molten-salt reactions toward the increased O chemical potential regime that favors the unstable (012) facet, perhaps exacerbating the poor cycle life of these already unstable compositions. However, in an all-solid-state battery, improved rate capabilities and cycle retention have been observed in SC CAMs with exposed (012) facets, which provide facile 3D ionic transfer channels from the surface to bulk.^163^ The effect of dominant facets on the electrochemical performance of SC CAMs has also been demonstrated by, Lu et al. as described previously,^149^ where both (012)-octahedral and (104)-polyhedral SC-NMC811 were synthesized by using NMC811-hydroxide and NMC811-carbonate precursors, respectively, lithiated under identical conditions. The hydroxide precursor was obtained through the conventional hydroxide coprecipitation route while the carbonate precursor was produced via a hydrothermal route. This emphasizes the importance of precursor choice on the final material properties including exposure of particular facets for improved electrochemical performance.

### Scale Up

The lab-scale synthesis of SC cathode materials allows for a great deal of flexibility regarding reaction conditions and processing; however any viable cathode material must be compatible with scale-up processes. Since SC-LiTMO_2_ materials are already available from a variety of CAM manufacturers, even including materials with a Ni content exceeding 90%, the feasibility of scale-up is confirmed. While the current LiTMO_2_ cathode production infrastructure (for PC and SC materials) supports the well-known hydroxide coprecipitation and traditional solid-state routes, others such as molten-salt may be more difficult to scale-up. Further, the SC particle size should be considered, as smaller particles approaching the submicron level, are often more difficult to handle and process. This is especially important on moving to Ni-rich compositions, where large SC particles are more difficult to grow.

Because Ni-rich cathodes require an O_2_-rich atmosphere, a tube furnace is a commonly used batch-processing method. In this setup, precursors are placed in a stationary quartz or alumina tube while an O_2_ flow is continuously supplied to maintain the required oxidation conditions. In transitioning from batch to continuous processing, a rotary tube furnace offers advantages by continuously rotating the sample, thereby enhancing heat and mass transfer, leading to a more uniform particle size distribution and improved crystallinity. Additionally, the furnace is sloped to facilitate the controlled movement of the powder, allowing the synthesized material to exit postreaction. However, the rotary tube furnace is incompatible with molten salt synthesis due to the formation of a highly viscous flux mixture during calcination, which adheres to the reactor walls once it solidifies postreaction and disrupts continuous operation.

That notwithstanding, larger-scale synthesis is possible and has been demonstrated by Qian et al., where a 2 kg batch of SC-NMC622 was produced in a furnace via molten salt using a LiOH/Li_2_SO_4_ flux,^166^ although this is still a small quantity considering that an estimated 175 tonnes of NMC622 CAM are needed to deliver just 100 MWh of storage capacity, based on typical cell-level energy densities and material fractions. Challenges associated with the molten salt approach are presented in the extra costs associated with the additional salts, recovery of salts postreaction, the tedious nature of the procedure, and the fact that after calcination/sintering the powder is often compacted into dense “brick” like chunks, difficult to remove from the crucible and which further require grinding, sieving, washing and annealing.^124^ Such processes often render these types of syntheses inconsistent across batches and hinder the production capacity as existing infrastructure would require adjustments, with the possibility of new infrastructure required. Thus, nonmolten salt methods present an easier and most cost-effective route to scale-up of SC CAMs. In particular, the two-step lithiation type processes show particular promise in Ni-rich SC CAM production, as they are compatible with the existing infrastructure that exists for high TRL level CAM manufacture.

## Electrode Formulation and Manufacture

Once synthesized, the desired (CAM) must be incorporated into an electrode. To achieve this, it must first be mixed into a slurry. The standard cathode mixing process involves combining the CAM with conductive carbon, a polyvinylidene fluoride (PVDF) binder, and an *N*-methyl-2-pyrrolidone (NMP) solvent. The formulation of the slurry is the first step in a series of interconnected processes, each of which must be meticulously controlled and optimized to ensure optimal electrochemical performance.^167,168^ These steps, including mixing, coating, and calendering, collectively influence the chemical, mechanical, and electrochemical properties of the electrode and, by extension, the final electrochemical cell.^169^

The primary objective of the mixing process is to create a homogeneous, conductive, and sufficiently viscous slurry for use during coating and calendaring. This is typically achieved through a two-stage process: dry mixing with solid components, followed by wet mixing with PVDF and NMP. This is usually completed using high-shear mixers or planetary ball mills which are particularly effective at breaking apart particle agglomerates, a difficulty that becomes more pronounced with smaller particle sizes.^170^ During wet mixing, a solution of PVDF in NMP is added to suspend the solid particles. While one-step solvent addition has been reported, this approach generally results in inferior electrochemical performance compared to a series of sequential additions into the mixture.^169^ This has the additional benefit of high shear mixing to begin with, ensuring effective deagglomeration,^170,171^ followed by low shear mixing as the slurry viscosity decreases, preventing binder separation and delamination.^172^ Notably, single-crystalline CAM particles, owing to their uniform morphology and lower surface energy, tend to form more stable slurries and require less aggressive mixing conditions compared to their polycrystalline counterparts. Once mixed, the final volume of solvent is also important to consider. Adding too much can lead to particle coagulation, concentration gradients, and thin coatings, while adding too little can result in high yield stress and poor particle dispersion.^173^

For academic research to remain relevant in the ever-maturing Li-ion battery industry, research-grade electrodes must have mass loadings comparable to those used in the industry.^174–176^ In well-engineered commercial cells, electrode parameters such mass loading and porosity, and electrolyte parameters such as viscosity and ionic conductivity are already carefully optimized to ensure good electrode balancing, electrolyte wetting, and minimized salt concentration heterogeneity.^177,178^ Nevertheless, achieving these optimal electrode characteristics requires precise control over slurry rheology, which encompasses properties such as viscosity, shear thinning, and thixotropy, all of which influence the slurry’s behavior during processing and manufacturing.^179^

When NMP is used as a solvent, the conductive carbon additives in the slurry form a wet gel which flows under shear during coating.^180^ This gel serves to relay the adhesive properties of the binder to the CAM by forming bridges that link the ends of a polymer strand to the surfaces of two separate CAM particles. This creates a weakly coagulated state that stabilizes the structure and prevents particle aggregation.^181–183^ If the solid content is too low, then the disperse particles remain a low viscosity sol and do not gel with the binder. Whereas an optimal solid content enables the formation of a flocculation network that envelops the CAM particles and affords the best compromise between dispersion and viscosity.^184,185^

NMP is a reproductive toxicant whose use is restricted under regulation number 1907/2006 of the European Parliament and of the Council on the Registration, Evaluation, Authorisation, and Restriction of Chemicals (REACH). Consequently, there is a strong interest in reducing its use during the slurry mixing and coating process, not only for regulatory compliance but also to achieve higher mass loading. However, the shift toward aqueous or high solid content formulations is constrained by several factors. Aqueous solvents degrade the surface of Ni-rich cathode materials by forming surface Li_2_CO_3_, LiOH, and NiO species which increase the electrochemical impedance and polarization.^186^ Side reactions involving these species can also promote the dehydrofluorination of PVDF which leads to slurry gelation.^187^ Therefore, aqueous methods are not recommended without protective surface treatments, pH control, or alternative binders.^188–190^ Instead, high solid content or dry mixing is often preferred and is achieved through twin-screw extrusion with co- or counter-rotating screws.^191–194^

In dry mixing, the active material, conductive additives, and polymer binders are blended together under high shear forces without using a solvent. This removes the drying and solvent recovery steps which substantially reduces energy consumption and processing time.^195,196^ Additionally, the absence of solvent prevents the formation of drying-induced concentration gradients within the electrode, a process which can cause cracking or delamination in thicker electrodes produced by slurry methods.^197^

In dry mixing, the binder type is critical. Here, PTFE is often preferred due to its ability to fibrillate under shear.^198^ This allows it to form a robust fibrous network that binds the particles together.^199,200^ The high mechanical stress applied throughout dry mixing also plays a critical role in deagglomerating carbon black, thereby increasing homogeneity and enhancing electronic conductivity across the electrode.^201,202^ When combined with the technique’s ability to produce electrodes with high mass loading (>5 mAh cm^–2^),^203^ these advantages make dry mixing particularly attractive for scalable manufacturing of high-performance Li-ion battery electrodes.

Once slurry formulation is complete, several methods are available for coating and are briefly summarized in Figure 7. Academic studies have explored techniques such as spray coating,^204,205^ electrophoretic deposition,^206,207^ and 3D printing.^208–210^ However, these methods are limited by low mass loading rendering them unsuitable for industrial applications.^211^ As a result, draw-down and roll-to-roll methods are more commonly used due to their ability to produce thicker, denser films.^211–214^

**Figure 7:**
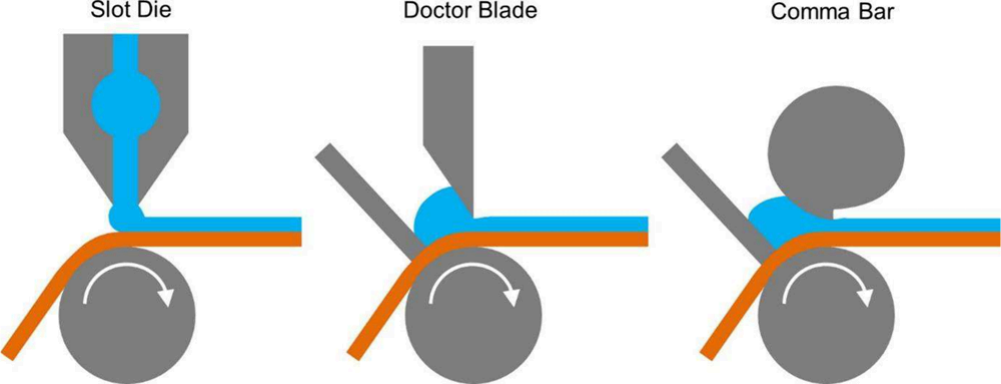
Illustration of slot die, doctor blade and comma bar coating geometries on a roll-to-roll coater. Reproduced with permission from ref 211. Copyright 2021 Elsevier. Licensed under CC-BY 4.0.

Dense, solvent-containing, slurries exhibit non-Newtonian properties.^215,216^ Therefore, to promote shear-thinning behavior, slurry coating is often conducted at elevated temperatures.^217^ One study observed that wet slurries coated at 60 °C exhibit 23% lower viscosity compared to those applied under standard conditions. Consequently, coating speed could be increased by ≈14% without a detrimental effect to electrode thickness.^218^ As with all methods, the thickness, porosity, and density of the final electrode are all properties that affect volumetric energy density. These variables are controlled by a process called calendering.^219^

Calendering is the final step in electrode manufacturing. Here, the dried electrode is compressed to achieve a desired thickness. This process increases particle contact and improves electronic conductivity.^220^ However, overcalendering can significantly reduce the void space, thereby impairing the wetting ability of the electrolyte which hinders Li-ion transport.^221,222^ Conversely, under-calendering results in poor interparticle contact which leads to resistive (ohmic) losses. As such, careful optimization of this manufacturing step is needed. Such optimization is also chemistry- and morphology-dependent. For example, Ni-rich cathodes, which are mechanically weaker than their low-Ni counterparts,^223^ may experience greater structural degradation when subjected to the same press densities.^219,224^ Compared to PC particles, SC particles can be calendared 33% more densely and still exhibit superior electrochemical performance without cracking.^219^ Together, this highlights the importance of balancing chemical and morphological design considerations when aiming to optimize electrode performance. While Ni-rich cathodes offer higher energy density, their mechanical fragility necessitates careful consideration of processing parameters to limit additional sources of mechanical degradation.

While robust SC morphologies allow for calendering to greater electrode densities without compromising electrochemical performance, there is a limit to the calendering force that can be applied. Excessive pressure can result in the embedment of CAM particles into the Al current collector. While this has been demonstrated to improve electrochemical performance (attributed to enhanced particle-foil contact and reduced polarization resistance) it also increases the risk of cathode delamination and current collector fracturing.^225,226^ This poses a significant challenge that must be overcome when optimizing calendering parameters in the pursuit of greater battery performance. Tailoring electrode architectures to the cathode’s chemical, morphological, and mechanical properties is essential for achieving both high energy density and good cycle stability.

## Electrochemical Performance

The total amount of energy that can be stored within an electrochemical cell is given by eq 1, where U represents energy density (Wh g^–1^), Q represents the total charge per unit weight (Ah g^–1^), E_cell_(q) represents the potential difference of the cell at a given state of charge (V), and dq is the infinitesimal amount of charge delivered at a given resolution over the interval from 0 to Q (Ah g^–1^).

$$U=\int_{0}^{Q}E_{cell}\left(q\right)dq$$ 1

From this, two variables arise that can be optimized to enhance energy density in a single electrochemical cell: capacity and voltage. Increasing capacity allows for more charge to be stored per unit weight, while increasing the voltage raises the amount of electric potential energy delivered per unit of charge. The theoretical specific capacity refers to the maximum number of electrons that can be transferred per unit mass of active electrode material; it is calculated using eq 2, where Q^th^ is the theoretical specific capacity (mAh g^–1^), n is the number of participating electrons (for Li^+^, n = 1), F is the Faraday constant (96485 C mol^–1^), and M_W_ is the molecular weight of the active material (g mol^–1^).

$$Q_{th}=\frac{n\times F}{3600\times M_{W}}$$ 2

A flaw in the determination of theoretical specific capacity lies in the inherent assumption that the delithiated end member is thermodynamically stable as it is for materials such as LiMn_2_O_4_ and LiFePO_4_. Acting as a calculated “maximum capacity,” this term (obviously) disregards the existence of deleterious reactions that trap Li during charge and prevent its reinsertion during discharge. For Ni-rich LiTMO_2_, with a NiO_2_-like end member, it is unlikely that 275 mAh g^–1^ can be reversibly cycled without first stabilizing the unstable R3̅m NiO_2_-like phase. As such, in LiTMO_2_ cathodes, the theoretical capacity has not yet been achieved. Instead, what is obtained experimentally is considered to be the practical capacity, and for NMC cathodes is qualitatively determined by the Ni content. Increasingly Ni-rich compositions enable proportionally greater capacity extraction in a given voltage window (Figure 8).^95,227–229^

**Figure 8:**
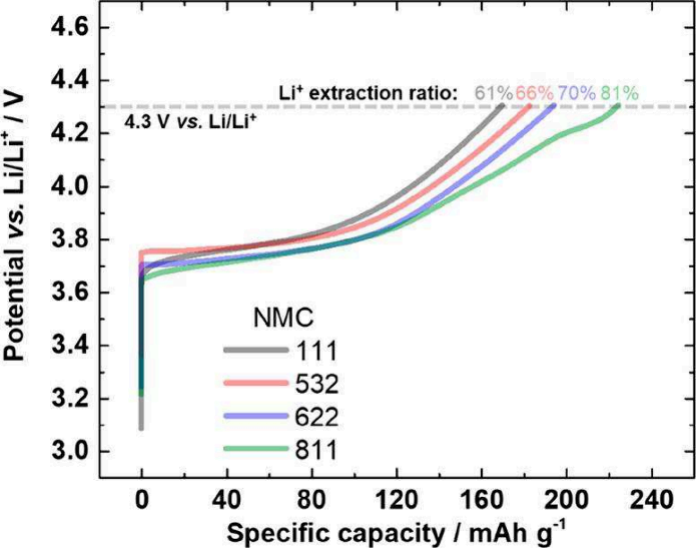
Electrode potential curves in the initial charge process for different NMC compositions using an upper cutoff potential of 4.3 V vs Li/Li^+^ and a specific current of 30 mA g^–1^. For each NMC composition, characteristic specific charge capacities, and thus Li^+^ extraction ratios, can be concluded when the electrodes are charged to equal cutoff potentials. Reproduced with permission from ref 229. Copyright 2019 American Chemical Society.

The voltage at which capacity is delivered can be defined by the difference in the chemical potential of both electrodes and is given by eq 3, where μ_*Li*_^*cathode*^ is the chemical potential of Li in the cathode, μ_*Li*_^*anode*^ is the chemical potential of Li in the anode.^230^ In electrochemical terms, it is technically correct to refer to the cathode as the negative electrode during charge (electrolytic) and the positive electrode during discharge (galvanic).^45^ However, a commonly used “battery convention” dictates that the cathode is always considered to be the positive electrode irrespective of the direction of the redox reactions taking place.

$$E_{cell}\left(q\right)=-\frac{\left(\mu_{Li}^{cathode}-\mu_{Li}^{anode}\right)}{n\times F}$$ 3

For LiTMO_2_ cathodes, the potential difference is not constant during electrochemical operation. During charge, the voltage steadily increases as the chemical potential of Li in the cathode rises, with the opposite being true for discharge. Thus, to maintain battery safety, the operation of a Li-ion battery is confined within an operational voltage window, which is bound by the depth of discharge (DOD) and upper cutoff voltage (UCV). Optimisation of these two variables requires consideration of material dependent effects occurring at both extremes.

Within the conventional cycling window of 3.0–4.2 V, NMC811 delivers a first cycle discharge capacity of ≈180 mAh g^–1^, at an average discharge voltage of ≈3.8 V vs Li^+^/Li. One study showed that increasing the UCV to 4.3 and 4.4 V results in a 9.5% and 14% increase in first cycle discharge capacity, respectively.^231^ Thereafter, the capacity fade in subsequent cycles is strongest in the highest UCV (Figure 9a). After 20 cycles, the 4.4 V UCV cell performs worse than the 4.3 V cell, and after 45 cycles, it performs worse than the 4.2 V cell. However, this performance fade is unrealistic due to the half-cell architecture, where an effectively infinite amount of Li is supplied by the Li metal anode.^232^ Instead, in a full cell (vs graphite), the performance of the 4.4 V UCV falls below the 4.3 and 4.2 V cells immediately after the second cycle (Figure 9b). These findings demonstrate that while higher UCVs can temporarily increase capacity, they significantly accelerate degradation, especially in full-cell configurations.

**Figure 9:**
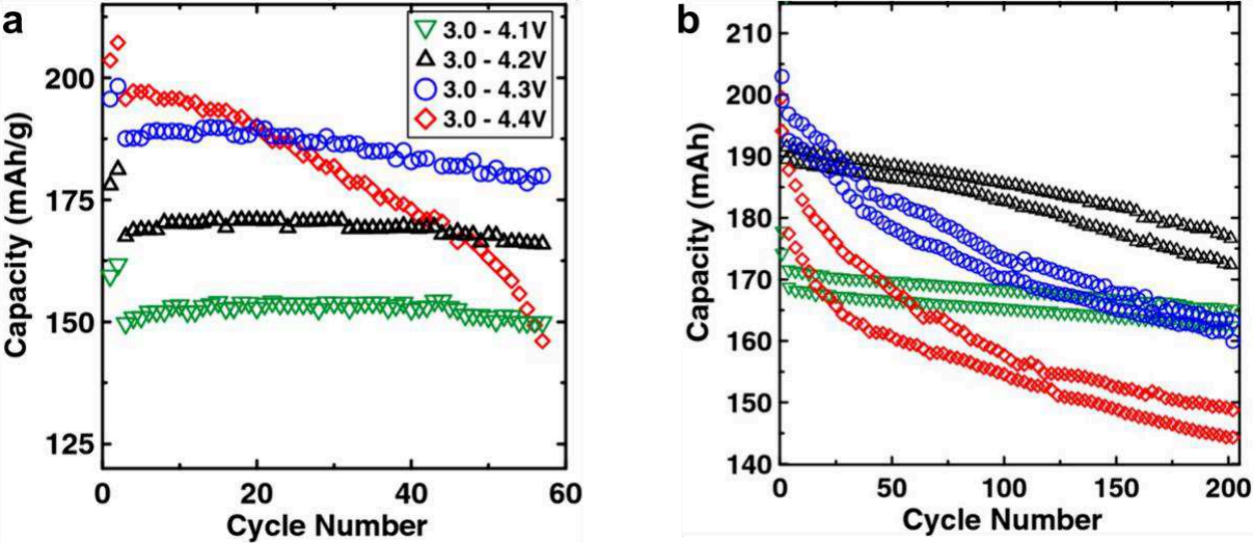
(a) Discharge capacity of NMC811/Li coin cells with 1 M LiPF_6_ in 3:7 v:v ethylene carbonate (EC):ethyl methyl carbonate (EMC) electrolyte as a function of cycle number for four different potential ranges. The first two cycles were cycled at C/20 with subsequent cycling at C/5. (b) Capacity of 2% VC graphite full cells as a function of cycle number for 200 cycles at a rate of C/5 in a temperature box at 30 °C. Adapted with permission from ref 231. Copyright 2015 The Electrochemical Society. Licensed under CC-BY 4.0.

Similar findings have been observed in other studies, where the effect of multiple temperatures is investigated. Here, capacity retention over 500 cycles was 91% and 92% for the 4.2 V cell at 25 and 40 °C, respectively, and 74% and 86% for the 4.3 V cell. This not only indicates worse capacity retention at UCVs exceeding 4.2 V vs Li^+^/Li for NMC811 but also highlights the nondeleterious effects of raising the cycling temperature to 40 °C within each voltage window. This work attributes the comparatively better performance of the higher temperature cells to increased Li-ion kinetics, reduced polarization, and a sacrificial surface dopant that was present on the surface of the SC-NMC811 particles.^85^

Difficulties in the scale-up of SC Ni-rich LiTMO_2_ cathodes has hindered research on the effects of long-term cycling under industry-relevant full-cell conditions.^39^ Fortunately, studies conducted on PC Ni-rich LiTMO_2_ cathodes are somewhat transferable to SC morphologies because material-independent phenomena occur in both types of cell. Regarding, therefore, general electrochemical capacity fade, two types exist: irreversible (thermodynamics) and reversible (kinetic). Irreversible capacity loss can occur via the reaction of electrochemically active Li-ions with carbonaceous species in the electrolyte to form a solid-electrolyte interphase (SEI) layer on the anode surface.^233–235^ This phenomenon was described as an “electric double layer” in the original patent filings of Akira Yoshino, wherein the SEI was believed, and subsequently proven, to passivate against parasitic electrolyte side reactions, prevent cointercalation of solvent molecules, and inhibit the exfoliation of graphite layers.^236–240^ Similarly, a thin and dense cathode-electrolyte interphase (CEI) helps to promote fast-ion diffusion and limit TM dissolution whereas a thick or inhomogeneous CEI promotes interfacial resistance and capacity fade.^241–244^ The formation of these layers is inevitable because μ_*Li*_^*anode*^ and μ_*Li*_^*cathode*^ often lie outside the thermodynamic stability window (redox potentials) of the electrolyte.^245^ As such, formation cycles control these layers’ formation such that Li loss can be minimized. Considering the greater risk of Li-ion consumption at the anode, SEI formation receives more attention in the design of these protocols. Here, high temperatures, fast cycling rates, and small DODs are desirable for stable SEI formation.^246^ SEI growth occurs at low anode potentials (0.1–0.3 V vs Li^+^/Li), where the SEI helps prevent exfoliation of the graphite layers and passivates the anode surface against further electrolyte decomposition. However, this passivation exists as a consequence of Li consumption and the formation of organic and inorganic Li-containing species. This makes SEI formation one of the primary sources of irreversible capacity loss and long-term degradation in commercial Li-ion cells.^247,248^ In contrast, the CEI is formed at high cathode potentials (>4.0 V vs Li^+^/Li), where it serves to limit electrolyte oxidation and TM dissolution.^249^ It is important to note that species such as Li_2_CO_3_, often attributed to the CEI, decompose above ≈3.8 V vs Li^+^/Li,^250^ and are generally not observed at high voltages in well-controlled experiments. Instead, degradation at the cathode is more directly linked to O_2_ loss at high state-of-charge, exacerbated by EC oxidation. These processes are primary triggers for the formation of reduced surface layers, which are a dominant degradation mode in cells cycled to high UCVs. These mechanisms are clarified in more detail in a later section, but we note that they are largely distinct from the notion of a CEI acting like a stable, SEI-like, organic film.

Depending on the experimental conditions (e.g., during calendar aging) the SEI and CEI can thicken with cycling, continuing to consume electrochemically active Li-ions as they do so.^251–254^ Among the two interphases, SEI growth is significantly more detrimental than CEI formation during calendar aging, as it leads to greater irreversible Li consumption and electrode slippage, where the capacities of each electrode no longer match what was initially used to balance the cell. This shifts the real operational voltage window upward and causes the cathode to cycle between higher states of charge.^255,256^

This links to rate-dependent kinetic capacity fade. As has been shown for SC-NMC811, O loss and the formation of O vacancies occurs at high voltages (>4.3 V vs Li^+^/Li).^29,257^ This promotes the combustion of EC which drives cathode surface reconstruction. Then, at low DOD (<3.0 V vs Li^+^/Li) the mobility of the formed O vacancies is increased, leading to the densification of rock salt-like surface phases that create kinetic barriers for Li-ion diffusion.^29,30^ This is summarized in Figure 10.

**Figure 10:**
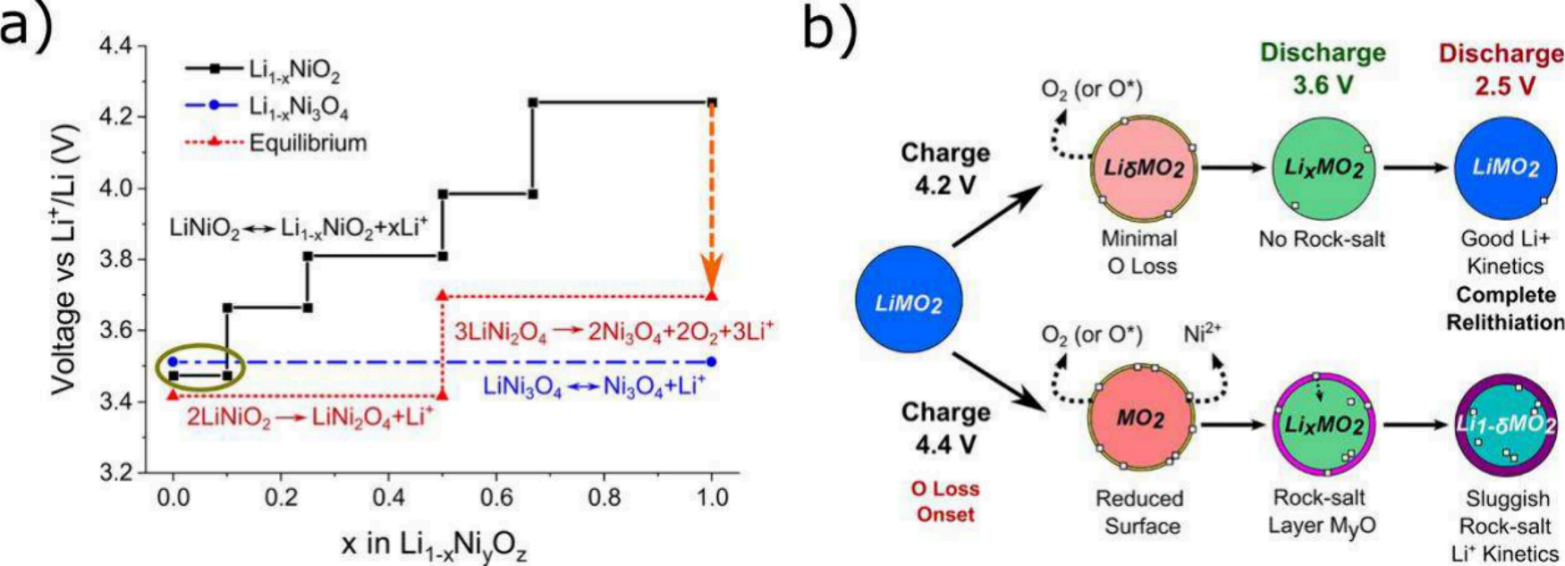
(a) Voltage vs degree of lithiation in Li_1–*x*_NiO_2_ predicted from DFT calculations. Black curve: layered Li_1–*x*_NiO_2_ structures. Red dotted curve: structural transformations between layered LiNiO_2_, spinel LiNi_2_O_4_, and Ni_3_O_4_ structures. Blue dot-dash curve: lithiation of the Li_1–*x*_Ni_3_O_4_ structure. Orange arrow: voltage difference between the layered structure pathway and the equilibrium pathway. Gold oval: The lithiation of the Ni_3_O_4_ phase is thermodynamically more favorable than the layered Li_1–*x*_NiO_2_ phase. (b) Schematic representation of the underlying mechanisms that govern synergistic degradation in Ni-rich TM oxide cathodes. Reproduced with permission from ref 29. Copyright 2023 American Chemical Society.

Altogether, LiTMO_2_ cathodes have now reached a sufficiently mature stage where the underlying chemistry and fundamental operating mechanisms are reasonably well understood. As a result, the optimization of their electrochemical performance now focuses on narrowing the gap between theoretical and practical capacities.^258^ Predominantly, this is achieved through a range of degradation mitigation techniques. This is evident throughout the literature, where studies investigating the electrochemical performance of cathode materials, be they SC-NMC811 or others, seldom focus solely on electrochemical performance, but instead on the amelioration of one or more degradation mechanisms through various means. However, in order to be assured of their efficacy, a thorough understanding of the degradation mechanisms they aim to target is necessary to obtain. Hence, the subsequent section will aim to summarize those mechanisms, their onset, severity, and impact on electrochemical performance.

## Degradation Mechanisms

The degradation of Ni-rich layered cathodes arises through a number of mechanisms that collectively span the bulk, surface, and interface. These encompass mechanical (e.g., fracture, plane gliding), chemical (e.g., oxygen loss, phase transitions), and electrochemical (e.g., surface reactions, Li trapping) reactions. This section outlines these degradation mechanisms by beginning with bulk structural changes and progressing toward surface and interface phenomena which ultimately limit the electrochemical performance.

### Changes in Bulk Crystallographic Structure

Within the bulk, mechanical degradation may result from crystallographic transformations and internal stresses which compromise structural integrity. Fortunately, in SC-NMC811, the deleterious effects of the H1, M, H2, and H3 transitions are mitigated by a disruption to long-range order. Consequently, the mechanical stress generated by these transitions is not as effectively transmitted to the primary particles in which it occurs. Therefore, SC cathodes are generally considered to be resistant to cracking.^87,259^ However, exceptions arise at high Ni content and under high UCV charging conditions.^102,166^

Due to the long Li-ion diffusion path length inherent to large SC particles, Li heterogeneity can develop during high C-rate operation. This results in a coexistence of Li-rich and Li-poor regions within individual SC particles.^260^ Similar to the effects of *c*-lattice collapse, the application of high current densities can induce internal stress, leading to structural defects such as microfractures.^261–263^ In SC particles, the formation of cracks along the (003) direction can be simulated using a strain model which, unlike those in polycrystalline (PC) morphologies, do not result in particle pulverisation.^97,264^ The stress induced by the Li-ion concentration gradients has also been shown to cause plane gliding (Figure 11), where scanning electron microscopy (SEM), studies observed surface cracks perpendicular to the (003) direction on particles cycled to 4.3 and 4.4 V vs Li^+^/Li for 200 cycles.^36^ A recent study on SC-NMC811 revealed that the origin of this plane gliding arises due to a 75% reduction in shear strength during cathode delithiation.^265^ These findings indicate that even mild mechanical loading can induce particle slippage and fracturing. The shear stress induced from this slippage is markedly different from *c*-lattice collapse; scanning transmission electron microscopy (STEM) analysis reveals that the *d*-spacing of the (003) plane remains unchanged in the layered structure before and after plane gliding occurs.^36^ When the UCV is increased to 4.8 V vs Li^+^/Li, the resulting microfractures permeate into the subsurface/bulk, facilitating deleterious electrolyte side reactions. Interestingly, reversibility is observed, and upon discharge to 2.7 V vs Li^+^/Li, the glide lines and microcracks disappear. This is considered a direct observation of lattice-invariant shear,^266^ and is predicted to occur ubiquitously, irrespective of Ni content and morphology.^267^ It is thus worth noting that plane gliding is perhaps most clearly observed in Ni-rich cathodes due to the higher states of delithiation that can obtained at realistic UCVs, and in SC morphologies, due to the absence of buried grain boundaries that might otherwise obscure the effects of surface-localized stress.

**Figure 11:**
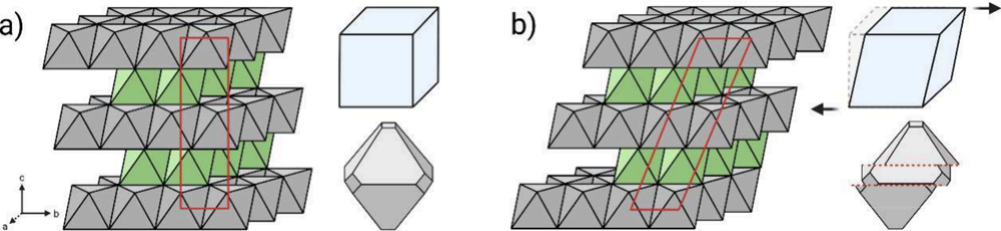
Illustration outlining the effects of plane gliding on the TM oxide crystal structure and SC particle.

Recent studies indicate that plane gliding is sensitive to the number of O vacancies which control the kinetic energy barrier for TM migration.^268,269^ It is shown that SC Ni-rich particles with fewer O vacancies exhibit less plane gliding and superior electrochemical performance.^270^ Therefore, to construct mechanically resistant single crystals, good control of the O sublattice is required, as it notoriously degrades, especially at the surface, during high-voltage cycling.

### Oxygen Loss and Surface Reconstruction

At the particle surface, chemical degradation becomes dominant through O loss and surface reconstruction which compromises interfacial stability. Unsurprisingly, the extent of surface O loss is material dependent. For layered LiTMO_2_ cathodes, the onset potential decreases as the Ni content increases, dropping from 4.6 V for NMC111 to 4.3 V for NMC811, and further to 4.1 V for LiNiO_2_.^257,271–273^ Typically, O loss is contextualised using a rudimentary understanding of the materials’ electronic structure. Here, the redox-active TM^3+/4+^ band is believed to overlap with the O^2–^ band, thereby leading to O oxidation and O_2_ evolution when electron density is consumed beyond a critical state of charge.^274^

However, “the concept of oxidation state is not particularly helpful when considering predominantly covalent compounds”.^275^ This is immediately evident when identifying the inconsistencies afforded by the ionic model. According to this theory, increasingly Ni-rich cathodes should exhibit less severe O loss due to the greater reliance on the Ni^3+/4+^ redox couple which is shown to not overlap with the O^2–^ band.^274,276^ Yet, the aforementioned experimental observations, together with the higher O_2_ loss onset potential of LiCoO_2_ (4.6 V vs Li^+^/Li) relative to NMC811, contradict this prediction.^277^ Moreover, spinel Li_1–*x*_Ni_0.5_Mn_1.5_O_4_, which operates on the same Ni^3+/4+^ redox couple as the layered oxides when 0.5 ≤ *x* ≤ 1, does not exhibit any O loss even at 5.0 V vs Li^+^/Li.^257^ Evidently, additional considerations are needed to identify its origin.

All R3̅m Li_(1‑x)_TMO_2_ cathodes (where x ≠ 0) are thermodynamically unstable. In the absence of kinetic barriers, phase transformations would occur spontaneously. The composition of the resultant phase(s) and the rate at which they form depend on the TM. Hence, to understand the thermal stability of NMC cathodes, ternary Li-TM-O phase diagrams involving all three NMC components (Mn, Co, and Ni) are considered. This helps to identify their respective roles in driving O_2_ loss.

Traditionally, Mn is incorporated into NMC cathodes to enhance thermal stability;^280^ thus, its role in promoting O_2_ loss should be low. Considering, therefore, the delithiation of layered Li_(1‑x)_MnO_2_, an irreversible rapid transition toward spinel LiMn_2_O_4_ occurs (Fd3̅m) at x = 0.5.^281–285^ Subsequent delithiation of the spinel phase, as illustrated by Figure 12a and the inset Figure 12b, occurs under equilibrium conditions along the tie line connecting LiMn_2_O_4_ and β-MnO_2_ (*C*2/*m*).^278^ Thus, Li_2_MnO_4_ (and by extension, Li_(1‑x)_MnO_2_) is not thermodynamically prone to O_2_ loss upon delithiation.

**Figure 12:**
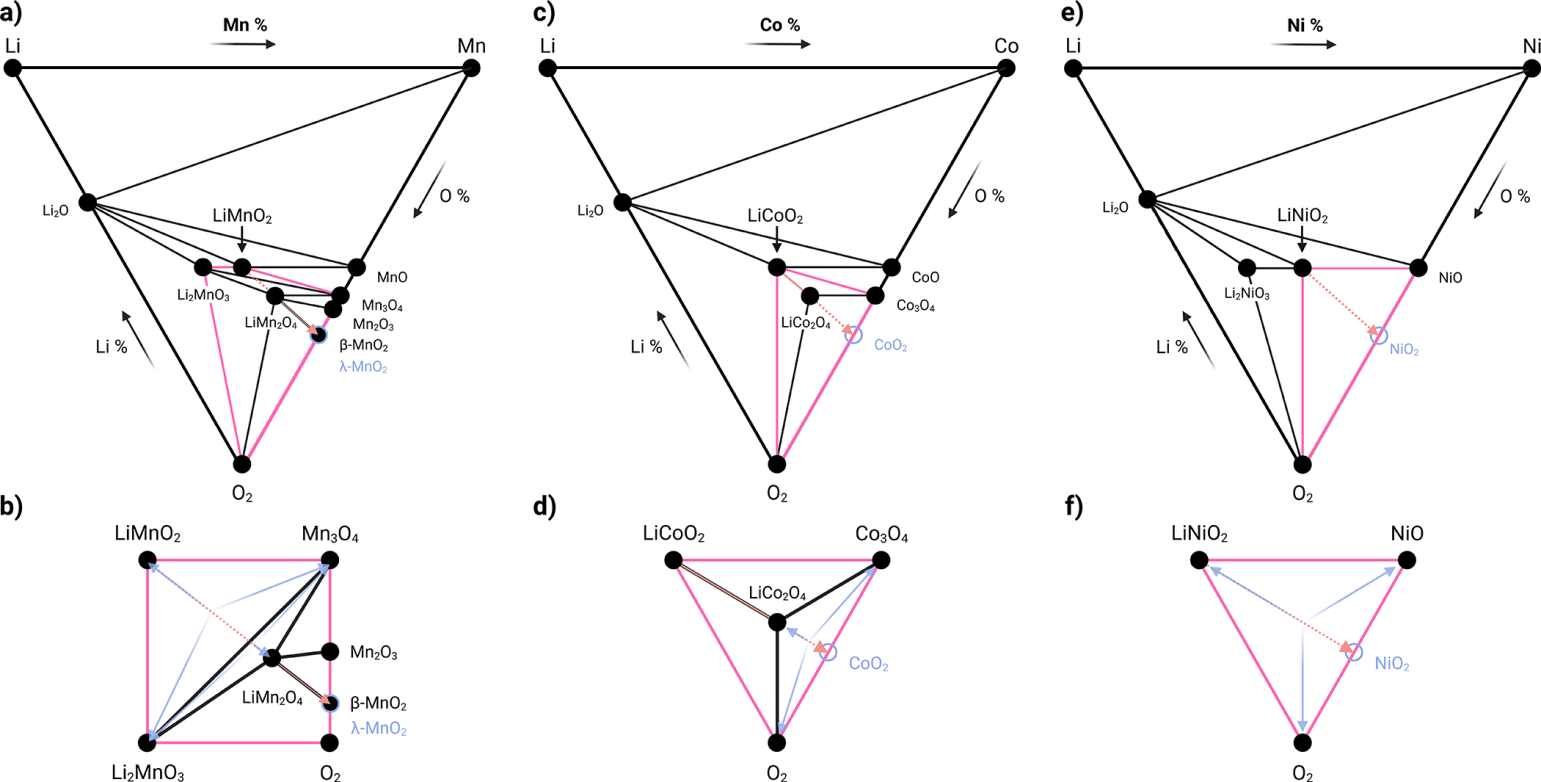
Ternary Li–TM–O phase diagrams illustrating the delithiation pathway of (a, b) LiMnO_2_, (c, d) LiCoO_2_ and (e, f) LiNiO_2_. Solid black circles indicate stable phases, while hollow blue circles indicate unstable phases. Solid black lines represent thermodynamic equilibrium. Dashed red lines indicate unstable delithiation, whereas solid red lines indicate stable delithiation. Blue arrows depict the thermodynamic driving force to form three more stable ternary phases. Adapted with permission from refs 278 and 279. Copyright 2006 American Chemical Society and 2023 IOP Publishing, respectively. Ref 279 is licensed under CC-BY 4.0.

Interestingly, the spinel LiTM_2_O_4_ structure is the lowest energy configuration for Li_0.5_TMO_2_ among all layered 3d TM oxides.^285^ However, for Li_(1‑x)_CoO_2_, while a thermodynamically stable LiCo_2_O_4_ structure is predicted in some instances,^286^ its formation from a layered phase has only been observed at extremely small length scales.^287^ Instead, under standard conditions, the limited kinetics of the available ions only enables an order/disorder transition at *x* = 0.5.^54^ Thereafter, as shown in Figure 12c and Figure 12d, the delithiation pathway cuts through the LiCo_2_O_4_–Co_3_O_4_–O_2_ triangle. Unlike the Li–Mn–O system, the delithiated endmember (R3̅m CoO_2_) is unstable; therefore, no equilibrium exists. As a result, a thermodynamic driving force arises that promotes the formation of three (more stable) terminal phases: LiCo_2_O_4_, Co_3_O_4_, and, critically, O_2_.^278^

For Li_(1‑x)_NiO_2_, reconstruction toward spinel LiNi_2_O_4_ (Fd3̅m) is rare.^288,289^ Rather than being limited by kinetics, the layered-to-spinel transition requires an activation energy sufficient to make the LiNi_2_O_4_ phase thermodynamically unstable.^290^ Considering that Ni_3_O_4_ (Fd3̅m) is also unstable,^290,291^ the entire delithiation pathway of Li_(1‑x)_NiO_2_ (0 ≤ *x* ≤ 1) is unstable with respect to LiNiO_2_, NiO (Fm3̅m) and O_2_.^278^ This is shown in Figure 12e and Figure 12f.

Unlike simple ionic descriptions, thermodynamic phase stabilities clearly explain why increasingly Ni-rich LiTMO_2_ cathodes exhibit greater O_2_ loss than more Mn-rich compositions. This perspective suggests that Co- and Ni-based systems exhibit O loss due to their thermodynamic propensity to form unstable partially delithiated phases during delithiation, the decomposition of which leads to the formation of reduced phases and O_2_. This is supported by experimental observations of the terminal Co_3_O_4_ and NiO phases at the surfaces of delithiated LiCoO_2_,^292–298^ and LiNiO_2_,^32,131,299–307^ respectively. Stoichiometrically, this also explains why both spinel and rock-salt phases are observed in NMC cathodes, with the latter being more pronounced in Ni-rich compositions.^308,309^ In search of a more accurate electronic explanation that also clarifies why O_2_ loss is more pronounced in Ni-rich compositions, the differences among all three TMs can be attributed to the increasing TM–O covalency from Mn to Ni,^27,310^ accompanied by a corresponding decrease in O vacancy formation energy.^311^

Recognizing the thermodynamic origin of O_2_ loss is important because its release at high states of charge is a critical factor in the thermal runaway behavior of commercial Li-ion cells. O_2_ loss is most pronounced at the particle surface, where parasitic side reactions with the electrolyte exacerbate degradation. Consequently, gas evolution in SC Ni-rich NMC cathodes is significantly lower compared to PC alternatives.^312^ The release of O_2_ is also facet-dependent, with the (012) and (104) surfaces exhibiting the highest reactivity, and the (001) surface exhibiting the lowest.^32,165,306,313^ This highlights the promise of SC cathodes. With precise morphological control and intrinsic mechanical tolerance, the exposure of additional surfaces during cycling is minimal. Furthermore, the slower diffusion of O vacancies in SC cathodes limits TM migration, inhibits phase transformation, prevents further O_2_ loss, and increases safety.^314^

Following O_2_ loss, electrolyte decomposition is a key contributor to the observed electrochemical performance fade and can occur via two main routes: (1) electrochemical oxidation at high potentials (e.g., > 4.95 V vs Li/Li^+^)^315^ or (2) chemical oxidation through a reaction with singlet O_2_ released from the cathode surface.^316–318^ Both pathways lead to the production of CO and CO_2_ gases, the onset of which is used as a proxy for the O_2_ loss threshold.^20,318,319^ While some results suggest that gas evolution arises solely from electrolyte oxidation at high voltage, the absence of CO_2_ and CO evolution up to 5.0 V vs Li^+^/Li in LNMO spinel cathodes indicates that RSL formation and gas evolution are more closely linked to O_2_ loss rather than to the electrochemical oxidation of the electrolyte.^257^ However, catalytically active double ligand hole sites at the Ni-rich LiTMO_2_ particle surface do appear to exacerbate this decomposition,^26^ and justifies why investigations into electrolyte stability should be conducted against realistic TM oxide counter electrodes.^320,321^ Within these cells, electrolytes containing EC are particularly detrimental to the performance of Ni-rich LiTMO_2_ cathodes, as they accelerate O_2_ loss at the cathode-electrolyte interphase.^29,322^ The subsequent exothermic chemical oxidation (combustion) of the electrolyte, exacerbates the thermal instability of the cathode, which in turn promotes O_2_ loss.^323^ This creates a self-reinforcing cycle whereby surface NiO-like phases densify with successive cycles.^29,324^

By way of a brief digression, O_2_ formation has also been observed in the bulk of Ni-rich LiTMO_2_ cathodes via RIXS analysis.^25,67,325^ Without the Li-rich stoichiometry required by the ionic model, signatures previously attributed to so-called “O-redox” were initially confusing. However, much like how defects at the particle surface have been shown to nucleate reductive phase transformations and O_2_ loss,^314,326–328^ those same defects are also computationally predicted to exist within the bulk of LiTMO_2_ particles.^75,311,329–333^ The resulting phase transitions in LiTMO_2_ cathodes are expected to occur over a short length scale, making them difficult to detect using long-range-sensitive techniques such as XRD. Therefore, to enable observation, it is necessary to study an inherently unstable material prone to significant reconstruction. Indeed, for delithiated Li_2_NiO_3_, rock salt domains have been observed using XRD and TEM,^334^ along with Ni reduction and bulk O_2_ RIXS signals.^335^ Similarly, many TEM studies have observed the formation of buried rock salt domains in stoichiometric LiTMO_2_ cathodes,^328,336–339^ and although no study has yet linked this phenomenon to the O_2_ RIXS signal, many have suggested that minimizing the number of defects would stabilize the O sublattice, reduce phase transitions, and prevent O_2_ formation.^340–344^ Based on these suggestions, it is hypothesized that such changes could, in turn, limit the observed O_2_ RIXS features, enhance structural stability and lead to improved electrochemical performance provided that equal states-of-charge are compared.

While admittedly less severe than in PC morphologies, O_2_ loss and the formation of reduced surface phases still occur in SC Ni-rich cathodes, where they continue to limit performance. These processes not only highlight the thermodynamic instability of Ni-rich systems but also establish their kinetic limitations, particularly the generation of trapped Li and the onset of crystallographic fatigue.

### Kinetically Trapped Li and Crystallographic Fatigue

The aforementioned surface and interface transformations contribute to electrochemical degradation via kinetic limitations and phase inhomogeneity. Surface reconstruction impedes the diffusion of Li-ions and leads to a gradual decline in capacity and rate capability, particularly in materials cycled to high and low UCV and DOD, respectively.^29^

Operando X-ray diffraction (OpXRD) is a powerful tool for studying the kinetics of structural evolution because changes in specific XRD reflections provide real-time insights into phase transitions and lattice parameter shifts. For NMC cathodes, the solid solution behavior offers an additional advantage: the *c*-lattice parameter can serve as a proxy for monitoring the state of charge, enabling precise tracking of structural changes during cycling. This capability is particularly useful for identifying the formation of inactive phases and understanding their impact on electrochemical performance.

As illustrated in Figure 13, a 100-cycled NMC811/graphite full cell from ref 30 demonstrates a significant reduction in capacity at a 1C rate compared to its initial performance postformation. The extent of relative capacity fade increases when the rate is raised from C/3 to 1C after cycling. Concurrent with these electrochemical observations, an invariant XRD peak at approximately 18.5° is detected. Interestingly, the capacity loss, along with the evolution of the *c*-lattice parameter, can be recovered during a subsequent slower cycle. This suggests that the capacity fade observed for Ni-rich NMC cathodes cycled between 2.5 and 4.4 V is predominantly kinetically controlled. Given that prior studies on the same electrodes rule out bulk structural changes as a contributing factor to the observed degradation,^22,25^ these findings underscore the detrimental impact of surface-related changes on cell performance.

**Figure 13:**
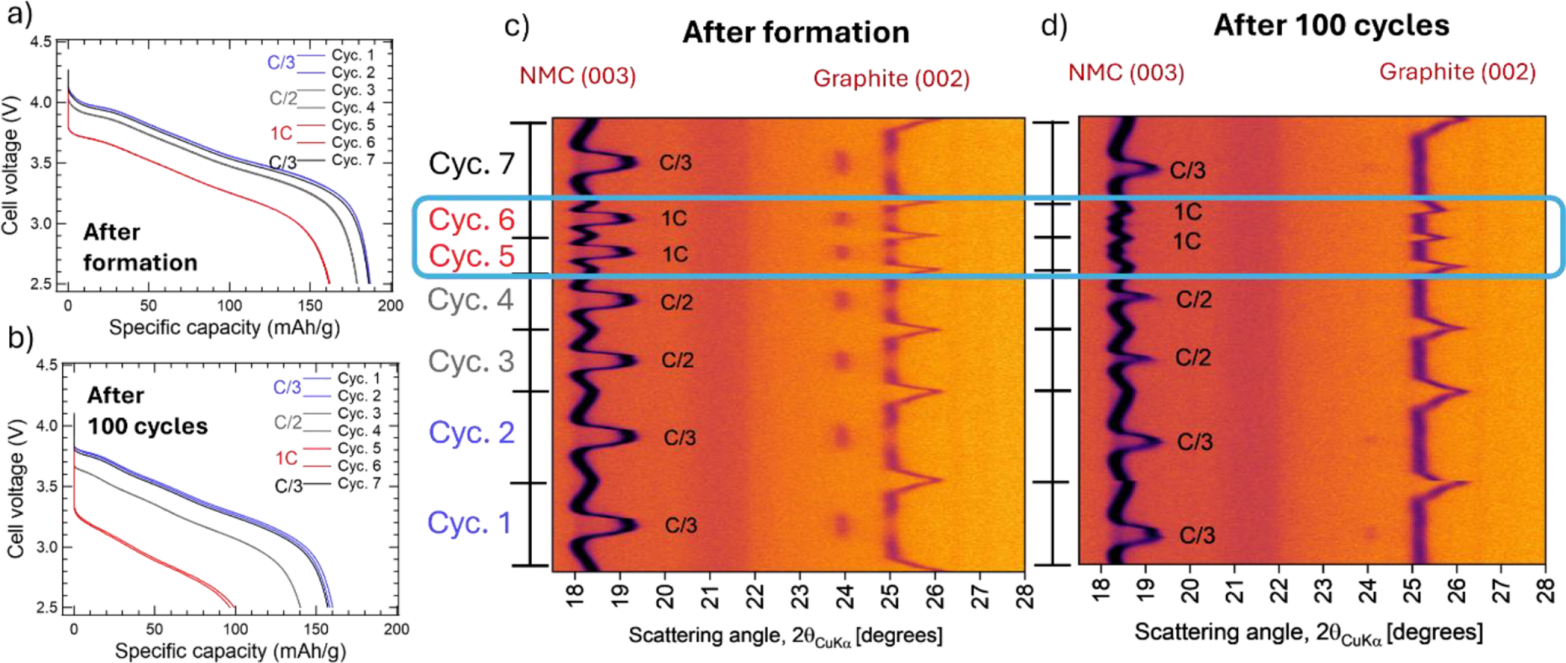
Electrochemistry data of the C-rate cycling protocol a) after formation and b) after one hundred cycles. Operando XRD during the C-rate cycling protocol c) after formation and d) after one hundred cycles to investigate Li ion insertion and deintercalation from the cathode to the anode and vice versa via the evolution of the NMC (003) and graphite (002) reflection. Blue square highlighting the *c*-lattice collapse due to continuous cathode delithiation and the lithiated phase (LiC_6_) at ≈ 24° of the anode electrode. Reproduced with permission from ref 30. Copyright 2025 American Chemical Society.

However, the exact mechanism by which surface reconstruction limits the Li-ion kinetics of Li is still an area of ongoing research. Therefore, it is necessary to consider the chemical and physical consequences of the RSL in Ni-rich LiTMO_2_ cathodes namely, crystallographic fatigue.

Crystallographic fatigue was first observed in an NMC811 cathode charged to 4.2 V vs Li^+^/Li, where one study reported a coexistence of “active”, “intermediate”, and “fatigued” phases.^345^ Similar results have been reported for other chemistries, including NMC333, NMC622, and NCA. For NMC333, a 5% spatial SoC variation could be detected using operando XRD.^346^ For NMC622, an inhomogeneous oxidation-state gradient could be observed in particles that were cycled to high voltages.^347^ For NCA, structural heterogeneity in the cycled cathode was evidenced by the presence of multiple layered R3̅m phases with different lattice parameters.^348^

It was initially proposed that approaching the O_2_ loss threshold increases interfacial lattice strain between the reconstructed rock-salt surface layer and the bulk layered structure.^345^ Consequently, this strain would restrict *c*-lattice expansion and thereby limit Li-ion diffusion. However, another study showed that despite the formation of a reconstructed surface layer, NMC811 could be cycled continuously to Li concentrations below 0.25 without any evidence of an additional ‘fatigued’ phase in the XRD data.^349^ This is surprising given the wide voltage range used (3.0–4.5 V vs Li^+^/Li) and was attributed to the hypothesis that fatigued phases may form only with extensive particle cracking. Supporting this, another study that used STEM imaging of cycled PC Ni-rich CAMs revealed that reduced surface layers predominantly form on open surfaces (exposed to the electrolyte), where the proportion of the fatigued phase increases as the battery ages.^350^

However, a comparison of electrochemical data recorded on SC vs polycrystalline agglomerates of LiNi_0.5_Mn_0.3_Co_0.2_O_2_ (NMC532) indicates that morphology does not appreciably affect the extent of kinetic limitations. Therefore, fatigue should be observable independent of particle morphology.^127^ On this, another study combined X-ray absorption spectroscopy with X-ray microscopy to spatially resolve a Ni oxidation state gradient near the surface of Ni-rich NCA primary particles (Figure 14).^351^ Here, the gradient extends deeper into the particle than the observed 1.5 nm rock salt phase and matches the initial description of fatigue wherein a layered structure is unable to reach the fully oxidized state. The same oxidation state gradient has also been observed in PC-LiNiO_2_ using STEM-EELS.^352^

**Figure 14:**
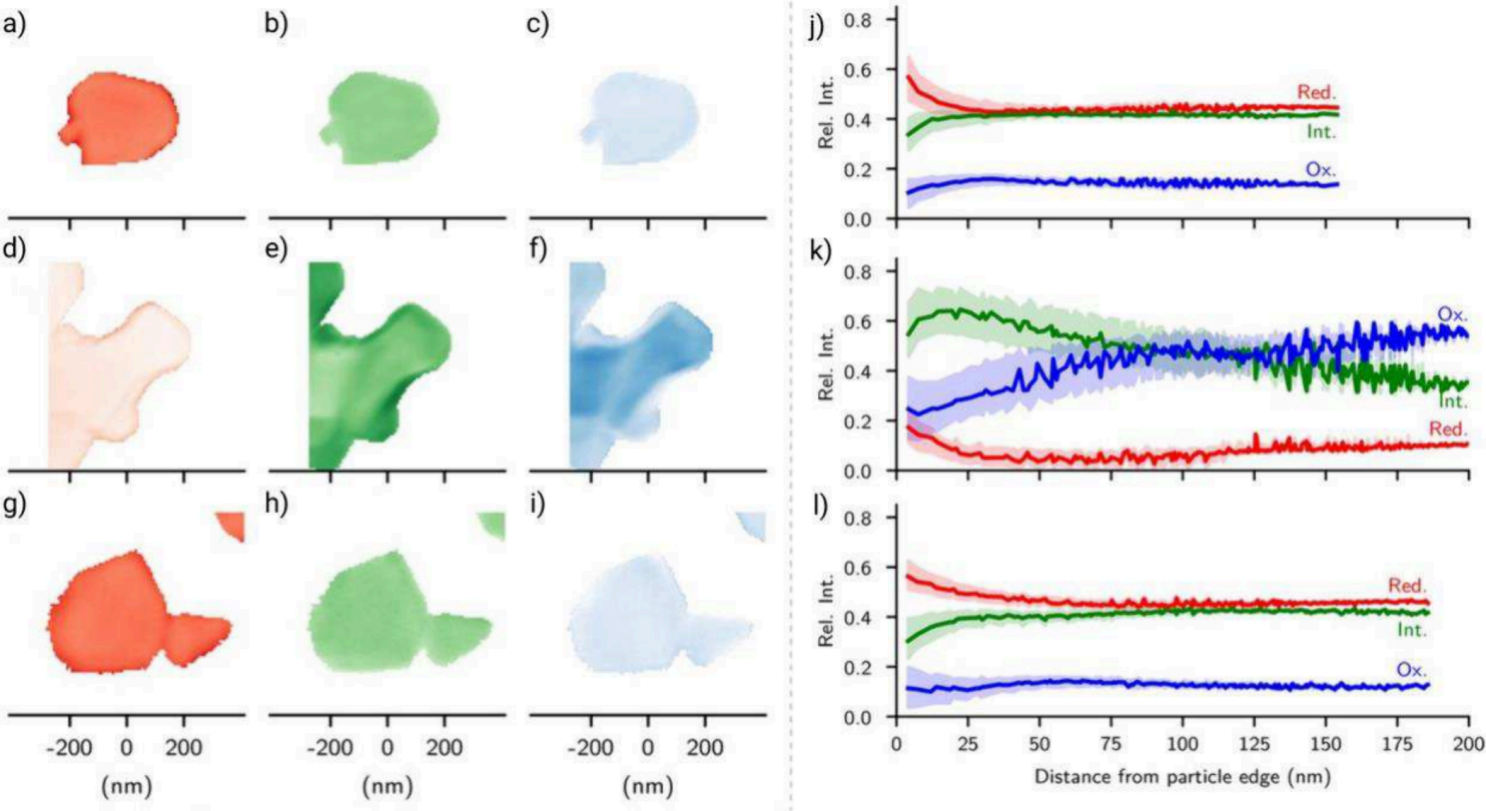
(a–c) Maps of Ni oxidation state for LiNi_0.8_Co_0.15_Al_0.05_O_2_ primary particles in the pristine state, (d–f) after charging to 4.75 V, and (g–i) after charging to 4.75 V, holding for 10 h, and discharging to 2.7 V at 60 °C. (j–l) Median proportion of Ni oxidation state segmented by distance from the particle edge for the same three conditions shown in (a–c), (d–f), and (g–i), respectively. Filled regions indicate one standard deviation above and below the mean. Adapted with permission from ref 351. Copyright 2020 American Chemical Society.

Returning therefore, to electrochemical considerations, one study observed that crystallographic fatigue appears most significantly at higher C-rates, with it diminishing during constant voltage (CV) holds and lower C-rate cycling.^353^ This is similar to ref 345, and continues to suggest that crystallographic fatigue is dominated by kinetic effects, limiting the rate at which a cathode can undergo delithiation.

The nature of the RSL is important to understand as its density and thickness can result in vastly different diffusive properties. It would be expected, as depicted in ref 83, that a stoichiometric NiO layer would be completely impermeable to Li-ion diffusion. However, identifying its exact chemical composition is difficult. Spectral observation of the RSL occurs via a peak in the O K-edge at ≈532 eV (Figure 15). However, DMFT calculations show that two different layers, NiO and Li_0.125_Ni_0.875_O, can be responsible for this feature.^30^ Consequently, it is difficult to experimentally validate the exact chemical composition of the RSL. However, considering that NiO exhibits no Li diffusivity, and that kinetic capacity fade appears to worsen in successive cycles, it is likely that the RSL thickens and densifies, i.e. tends toward NiO, with continued cycling at high UCV.

**Figure 15:**
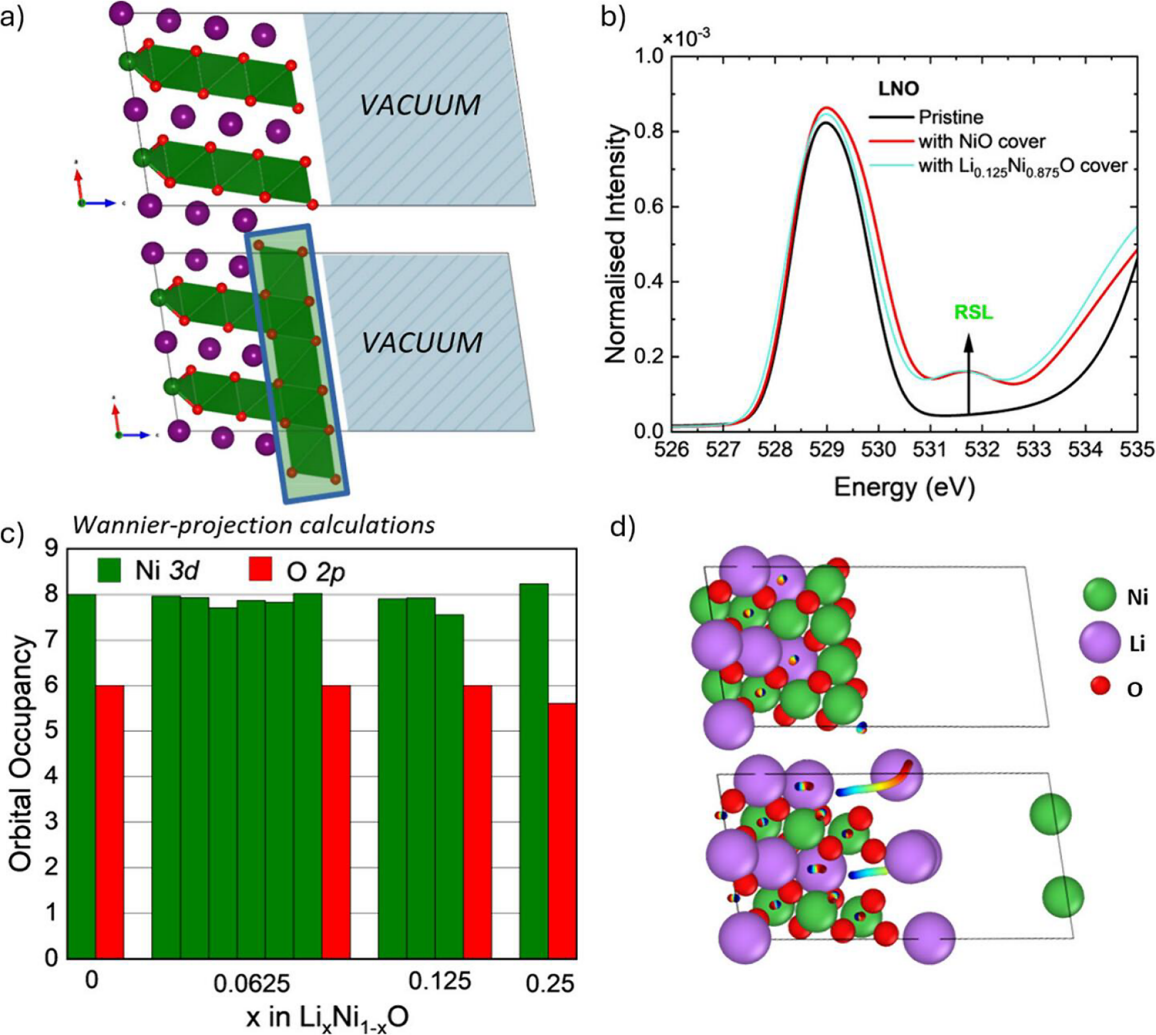
(a) Structural model of LiNiO_2_ with and without NiO on a chosen surface to simulate the RSL formation. (b) DFT calculations for the O K-edge XAS of LiNiO_2_ in its pristine form, with NiO and Li_0.125_Ni_0.875_O surface coverage. (c) Occupancies from Wannier projections from DMFTproj calculations are shown to explain the spectral similarities between the NiO and Li_0.125_Ni_0.875_O compounds. (d) Ab initio molecular dynamics (AIMD) calculations with an electric field applied in the *c* direction overlaid on the RSL end member structures. The rainbow lines correspond to trajectories in 250 fs of AIMD simulations and highlight how facilely the Li ions diffuse through the lithiated RSL member (bottom panel) compared to NiO (top panel). Reproduced with permission from ref 30. Copyright 2025 American Chemical Society.

Insofar as mentioned above, crystallographic fatigue and kinetic capacity fade can be considered the cumulative effect of structural and chemical changes that lead to mechanical degradation, particle heterogeneity, and O_2_ loss, all of which ultimately result in the kinetic isolation of otherwise thermodynamically available Li in the CAM. With these effects leading to performance fade under realistic operating conditions, a range of mitigation techniques have been suggested to alleviate their deleterious effects.

## Design Strategies and Future Outlook

Building upon the insights discussed above, future efforts toward enhancing long-term cathode performance must prioritise strategies that directly address intragranular cracking/microfracturing, surface reactivity, O_2_ loss, and rock salt layer formation. Among the most promising avenues are surface coating and bulk doping techniques, which have shown considerable potential in suppressing parasitic reactions and enhancing bulk stability.

### Doping

Doping involves the substitution of elements within a material’s bulk structure. In LiTMO_2_ cathodes, dopants are selected such that they preferentially occupy the Li *3b*, TM *3a*, or O *6c* sites. Ideally, the dopants should be stable once incorporated and should not be electrochemically active. Several elements—such as B,^354^ F,^355,356^ Na,^356^ Mg,^357–359^ Al,^357,358,360,361^ Ti,^359,362,363^ Fe,^359,361^ Y,^364,365^ Zr,^360,362,366–368^ Nb,^359,365,369–371^ Mo,^359^ Te,^363^ Tb,^372^ Ta,^373^ W^374^—have demonstrated the ability to enhance performance in SC Ni-rich layered oxide systems. While the examples above showcase doping effects across various SC systems, doping strategies are largely inherited from studies on polycrystalline materials. However, here the dopant mechanism of action should be specific to SC degradation modes. For example, while one particular mechanism of action is the ability to preserve the integrity of the bulk layered structure by creating robust metal-O bonds which may help to mitigate against O loss, another may be preventing plane gliding and limiting the formation of intragranular cracks through a pillaring effect.^359,375^ Further, the poor kinetics associated with the SC monolithic morphology may be improved through incorporation of large cationic dopants which can increase in the *c*-lattice parameter, effectively widening the Li layer, enhancing ionic diffusion, and mitigating the kinetically induced degradation that affects SC Ni-rich layered oxide cathodes in particular.

In one example, Ti- and Te-doped SC-LiNiO_2_ exhibited improved cycling stability compared to its undoped counterpart.^363^ Specifically, Te-doped LiNiO_2_ exhibited a capacity retention of 94% after 1C cycling between 2.8 and 4.4 V (vs Li^+^/Li). Here, gains were realized toward the end of discharge, a region particularly sensitive to lithiation kinetics.^376^ Therefore, it is not surprising that improved rate capabilities are also observed in Te-doped LiNiO_2_; it delivers a discharge capacity of 185 mA h g^–1^ at 5C (after a C/3 charge), an approximately 8% and 16% improvement versus uncoated LiNiO_2_ and Ti-doped LiNiO_2_, respectively.

Kinetic improvements in SC Ni-rich layered oxide cathodes have also been observed with Na doping at the Li *3b* site.^356^ DFT calculations showed that Na doping regulates the orientation of the Jahn–Teller distorted Ni-centered octahedra, thereby promoting the formation of a high-speed pathway for efficient Li-ion diffusion. This was demonstrated by the enhanced rate capabilities achieved up to 5C. Additionally, the Na-doped sample retained approximately 94% of its capacity after 100 cycles at 0.5C, compared to only 70% retention in the undoped sample.

Considering the success of single dopants, dual-doping has also become a popular strategy to improve electrochemical performance. Dual-doping aims to target distinct degradation processes, either through different mechanisms of action or by incorporating into various regions of the structure. For example, in dual Al/Zr-doped SC-LiNi_0.88_Co_0.09_Mn_0.03_O_2_, cycling stability was enhanced, with the Al dopant improving bulk Li^+^ diffusion and the less-soluble Zr dopant stabilized against rock-salt layer formation.^360^ Going beyond dual-doping, high-entropy materials e.g. LiNi_0.88_Mn_0.03_Mg_0.02_Fe_0.02_Ti_0.02_Mo_0.02_Nb_0.01_O_2_ combine many different mechanisms to improve performance.^359^ Here, Mg^2+^ acts as a pillar in the Li layer to prevent planar gliding, Fe^3+^ inhibits cation mixing, and Ti^4+^, Nb^5+^, and Mo^6+^ maintain structural integrity and prevent O_2_ loss. This high-entropy cathode material was able to exhibit 86% capacity retention after 3500 cycles between 2.8 and 4.3 V (vs graphite), and 91% capacity retention after 1000 cycles between 2.8 and 4.5 V (vs graphite).

### Coating

While doping strategies enhance the performance of cathode materials, additional protection is often required at high states of charge. To this end, surface coatings are applied to protect particle surfaces from deleterious reactions induced at high voltages, such as electrode–electrolyte reactions, surface reconstructions, CEI formation, transition metal dissolution, and HF attack.^39,377–379^ Examples of coating chemistries include oxides, sulfides, fluorides, phosphates, and organics.^377,380,381^

In one relevant example, a MnO_2_ coating was applied to the surface of SC-NMC811 by pretreating the hydroxide precursor with KMnO_4_.^382^ Here it was shown that a thin MnO_2_ layer (approximately 4 to 7 nm) reduces HF corrosion and suppresses TM dissolution at the electrode–electrolyte interface, resulting in improved Coulombic efficiency over 50 cycles at 0.1C. Li-containing coatings such as Li–S–O,^383^ Li_2_WO_4_,^384^ Li–B–O,^384^ LiNbO_3_,^385^ LiF,^386^ and Li_1.8_Sc_0.8_Ti_1.2_(PO_4_)_3_ (LTSP)^387^ have also been used as coatings for SC Ni-rich layered oxide cathodes. Additionally, coatings can be organic: for example, a poly­(3,4-ethylenedioxythiophene) (PEDOT) coating on SC-LiNi_0.83_Mn_0.1_Co_0.07_O_2_ has been applied by an oxidative chemical vapor deposition (oCVD) process to produce an ultraconformal surface layer (≈ 10 nm thick).^388^ Due to its ability to subdue CEI and impedance growth, the PEDOT-coated SC-NMC was able to exhibit 90% capacity retention after 200 cycles at 0.2C between 2.8 to 4.6 V (vs Li/Li^+^). This coating also resisted harsh calendar aging conditions, delivering a capacity retention of 85.3% after 100 cycles at 45 °C following a 72-h voltage hold at 4.6 V.

In aiming to improve the performance of candidate cathode materials, providing a physical barrier between the reactive cathode surface and the electrolyte mitigates surface mediated degradation. By doping the crystallographic structure, careful selection of dopant ions can selectively target bulk degradation known to affect the cathode chemistry in question. Altogether, as the trend toward ultrahigh Ni-rich compositions continues, adverse reactions such as O_2_ loss occur at progressively lower voltages. Therefore, advances in surface engineering and doping strategies become increasingly critical to pursue and understand.

### Particle Design

As discussed previously, tuning synthetic parameters to tailor the SC particle design (shape and size) is done primarily to improve structural and resulting electrochemical properties, mitigating against poor kinetics, surface reactivities and intragranular fracture. For example, by growing the particles such that the (012) facets are dominant, the kinetics were improved in SC NMC622 containing all-solid-state batteries, attributed to the (012) facet being capable of more facile 3D Li^+^ ion transfer.^163^ However, as the (012) facet has the highest surface energy, surface driven degradation may be exacerbated. The lowest energy (104) facet presents a more stable surface, and improved electrochemical performance has been demonstrated in (104)-dominant SC-NMC811 as described previously.^149^ Through particle size optimization, a balance must be struck between too small (aggravating surface instabilities) and too large (increasing the chance for intragranular fracture to occur). By employing a chemo-mechanical model, Pandurangi et al. posit that unconstrained SC NMC particles with size ≤ 2.5 μm should not undergo intragranular fracture up to cycling rates of 5C.^65^ Thus, well-designed SC CAM particles can inherently avoid cracking related degradation processes.

Altogether this review has provided a comprehensive overview of the many factors that influence the performance and longevity of SC Ni-rich LiTMO_2_ cathodes. By integrating insights from the atomic- and particle-level with practical considerations in their synthesis, formulation, and electrochemical behavior, the challenges associated with degradation and performance fade has been highlighted. A strong emphasis on morphological control and the deployment of experimental and computational characterization has underscored the critical need for a multiscale understanding of these materials. Strategies to mitigate degradation have been discussed and continued investigation into alternative coating techniques such as sol–gel, CVD, and atomic layer deposition can offer tailored surface chemistries with tunable thicknesses. Similarly, further research into doping techniques including solid-state diffusion, coprecipitation, and other solution-based methods, can enable more precise control over bulk properties, improve Li-ion diffusion kinetics, and reinforce structural integrity. As the demand for higher energy density intensifies, SC Ni-rich cathodes remain a promising platform to meet these targets. Moving forward, bridging the gap between academic fundamentals and practical implementation will be key to their successful integration into near- and next-generation Li-ion batteries.
